# Liquid Phase Exfoliation of 2D Materials and Its Electrochemical Applications in the Data-Driven Future

**DOI:** 10.1021/prechem.3c00119

**Published:** 2024-03-29

**Authors:** Panwad Chavalekvirat, Wisit Hirunpinyopas, Krittapong Deshsorn, Kulpavee Jitapunkul, Pawin Iamprasertkun

**Affiliations:** † School of Bio-Chemical Engineering and Technology, Sirindhorn International Institute of Technology, Thammasat University, Pathum Thani 12120, Thailand; ‡ Research Unit in Sustainable Electrochemical Intelligent, Thammasat University, Pathum Thani 12120, Thailand; § Department of Chemistry, Faculty of Science, Kasetsart University, Chatuchak, Bangkok 10900, Thailand; ∥ Department of Chemical Engineering, Faculty of Engineering, Kasetsart University, Bangkok 10900, Thailand

**Keywords:** Liquid Phase Exfoliation, 2D Materials, Transition Metal Dichalcogenides, TMDs, Size Selection, Electrochemistry, Energy Storage, Ionic Sieving, Machine Learning

## Abstract

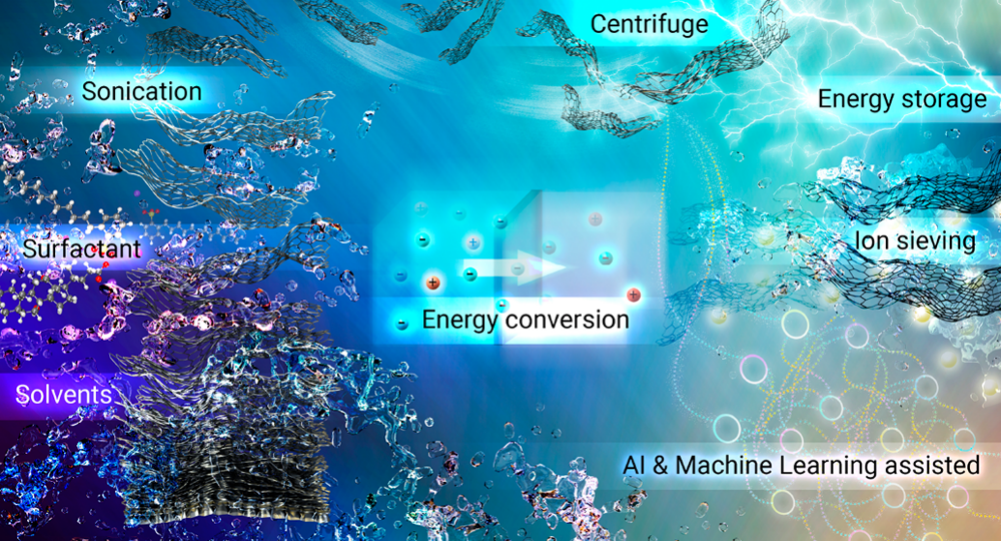

The electrochemical properties of 2D materials, particularly transition metal dichalcogenides (TMDs), hinge on their structural and chemical characteristics. To be practically viable, achieving large-scale, high-yield production is crucial, ensuring both quality and electrochemical suitability for applications in energy storage, electrocatalysis, and potential-based ionic sieving membranes. A prerequisite for success is a deep understanding of the synthesis process, forming a critical link between materials synthesis and electrochemical performance. This review extensively examines the liquid-phase exfoliation technique, providing insights into potential advancements and strategies to optimize the TMDs nanosheet yield while preserving their electrochemical attributes. The primary goal is to compile techniques for enhancing TMDs nanosheet yield through direct liquid-phase exfoliation, considering parameters like solvents, surfactants, centrifugation, and sonication dynamics. Beyond addressing the exfoliation yield, the review emphasizes the potential impact of these parameters on the structural and chemical properties of TMD nanosheets, highlighting their pivotal role in electrochemical applications. Acknowledging evolving research methodologies, the review explores integrating machine learning and data science as tools for understanding relationships and key characteristics. Envisioned to advance 2D material research, including the optimization of graphene, MXenes, and TMDs synthesis for electrochemical applications, this compilation charts a course toward data-driven techniques. By bridging experimental and machine learning approaches, it promises to reshape the landscape of knowledge in electrochemistry, offering a transformative resource for the academic community.

## 1 Introduction

There has been significant interest in two-dimensional (2D) layered materials, particularly since the breakthrough discovery of graphene in 2004 by A. K. Geim and K.S. Novoselov.^1^ These materials have attracted attention due to their ability to exhibit a wide range of characteristics, including high carrier mobilities,^2−4^ effective thermal and electrical conductivity,^5−7^ excellent mechanical strength,^8−10^ optical^11−13^ and catalytic properties,^14−16^ energy storage capabilities,^17,18^ and a variety of other characteristics.^2,19,20^ These unique properties make graphene well-suited for a wide range of uses, ranging from nanoelectronics to applications in the field of electrochemistry.^21−23^ Following the remarkable success of graphene, alternative two-dimensional (2D) materials capable of forming exceptional microscopic layers have had an equally astonishing surge in development. Transition metal dichalcogenides (TMDs) are examples of graphene alternatives to generate significant scientific study efforts because they are almost as thin, translucent, and analogous to graphene.^24^ Unlike graphene, which provides a fixed band gap, TMDs like MoS_2_, WS_2_, and WTe_2_ are natural semiconductors with a tunable bandgap.^11,25^ They are abundant in nature and can be transformed into ultrasmall, low-power transistors that offer greater efficiency compared to current silicon-based transistors, which struggle to cope with the rapid miniaturization of devices.^26,27^ The electrochemical properties of TMDs vary with their dimension, making monolayer or few-layered TMDs desirable for various electrochemical applications. These include but are not limited to energy storage,^28,29^ electrocatalysis,^30^ electrochemical sensors,^31,32^ and the development of ion-sieving membranes^33^ and electrochemical adsorption systems.^34^ For instance, specific monolayer TMDs with semiconducting properties also exhibit visible-region photoluminescence.^35^ These attributes position them as potential candidates for applications in optoelectronics^36−38^ and sensing.^39,40^

**Figure 1:**
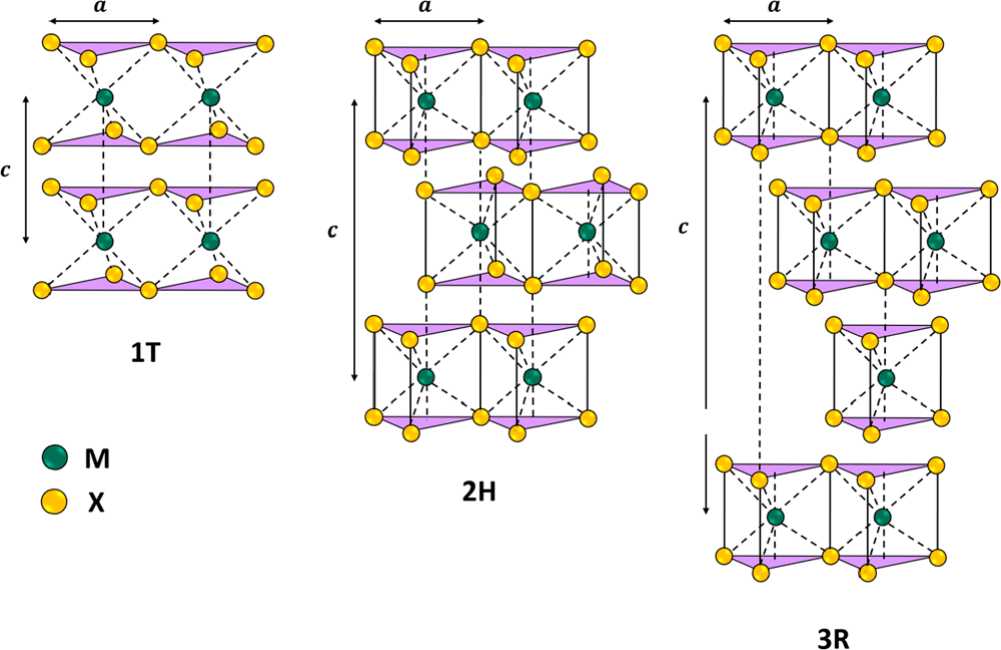
Schematic representations of the structural polytypes: 1T (tetragonal symmetry, one layer per repeating unit), 2H (hexagonal symmetry, two layers per repeating unit), and 3R (rhombohedral symmetry, three layers per repeating unit).

Polymorphism stands out as a distinctive characteristic of transition metal dichalcogenides, and they typically exhibit hexagonal symmetry in their common polytype. These materials adopt a layered structure with three potential stacking configurations, identified as 1T, 2H, and 3R, as illustrated in Figure 1. These labels correspond to trigonal, hexagonal, and rhombohedral arrangements, with the numerical value signifying the quantity of X-M-X units within the unit cell.^41,42^ TMDs consist of metal atoms M (typically from groups IV to VII) sandwiched between two layers of chalcogen atoms X (usually S, Se, or Te), with the chemical formula MX_2_.^43,44^ The atoms within the layers are held together by strong covalent bonds, while the layers are connected by relatively weak van der Waals (vdW) forces.^45^ To produce mono- or few-layer TMDs nanosheets from bulk powders, various techniques were developed to overcome the weak van der Waals interaction between adjacent layers. These methods can be categorized broadly into top-down and bottom-up approaches.

Bottom-up methods involve cultivating layered nanomaterials in specific environments using atoms or molecules as building blocks, which include techniques like chemical vapor deposition (CVD), physical vapor deposition (PVD), hydrothermal, and solvothermal processes.^42,43^ The chemical vapor deposition (CVD) technique is a representative bottom-up approach frequently employed in the fabrication of 2D nanomaterials. This method is capable of generating defect-free, large lateral size monolayers of graphene,^46−49^ TMDs,^50−53^ and hexagonal boron nitride (hBN)^54−57^ with high crystallinity. Additionally, there have been theoretical explorations of the potential of CVD in synthesizing emerging 2D materials like silicene,^58−60^ MXene,^61^ phosphorene,^62,63^ and borophene.^64^ However, successful implementation demands precise control of experimental parameters, given the multitude of influential factors throughout the process.^65^ Consequently, achieving large-scale production of high-performance, minimally defective monolayer and few-layer nanosheets remains a challenge.^66^ Hence, the complexity and cost associated with this method currently hinder its mass-scale production. Contrastingly, physical vapor deposition (PVD) involves applying intense energy to a pure solid material, leading to the formation of a vapor that can condense on a substrate surface within an ultrahigh vacuum setting.^67^ PVD facilitates the processing of high-purity 2D materials without inherent size restrictions, ensuring uniformity over extensive areas (exceeding 1 square meter^68^). This capability extends to various materials, including TMDs,^69,70^ graphene,^71,72^ hBN,^73,74^ and even encompasses borophene.^75^ Nevertheless, PVD comes with certain drawbacks, including constraints on material selection, the necessity for a high-vacuum environment (approximately 1 × 10^–8^ Torr), and a relatively slow growth rate.^76^ Moreover, the elevated energy linked to the incident particle flux can induce damage to the lattice, particularly in the context of graphene synthesis,^68,77^ thereby impeding its efficiency in the production of 2D materials. Hydro/solvothermal methods involve producing materials within sealed containers under elevated temperatures and pressures, conducted either in water (hydrothermal) or alternative nonaqueous solvents (solvothermal).^78,79^ These techniques provide substantial yield and enable the large-scale manufacturing of 2D materials at a comparably lower expense, offering potential suitability for industrial applications.^24^ However, achieving conclusive monolayer fabrication has not been definitively established.^36^ In summary, challenges in scaling production and maintaining precision, along with the associated complexity and costs, have diminished the preference for bottom-up methods in synthesizing few-layered 2D materials. Alternative synthesis methods are now more favorable for both the production of 2D materials and their utilization in various electrochemical applications.

Top-down methods concentrate on transforming bulk crystals and layered compounds into monolayer or few-layer 2D materials. These top-down techniques include mechanical, liquid, and electrochemical exfoliation.^42^ Mechanical exfoliation involves detaching individual layers or a small number of layers from bulk crystals using adhesive tape (“scotch” or “blue” tape).^80^ This process yields high-purity, pristine single-crystal flakes suitable for fundamental analysis and even the creation of individual devices.^36^ Nonetheless, this technique is not suitable for large-scale production. Liquid phase exfoliation (LPE) has been widely employed for the exfoliation of layered compounds into 2D nanomaterials.^81,82^ The LPE procedure can be carried out in two different methods, which are direct exfoliation (sonication/shearing-assisted) and ion intercalation. LPE stands out as a simple and highly appealing technique for generating solution-processed 2D materials. It is particularly suitable for applications demanding substantial quantities of 2D materials at a cost-effective rate, offering improved electronic quality when compared to mechanical exfoliation and chemical vapor deposition techniques.^83−86^ The LPE could produces large amount of MoS_2_ nanosheets (solid/suspension), which can facilitate the creation of thin films electrode using a variety of techniques e.g., filtration, inkjet printing, and spin-coating.^83,87−89^ While liquid-phase exfoliation (LPE) can yield significant quantities of nanosheets, a significant challenge lies in enhancing the production of monolayers and maintaining their lateral dimensions. Despite the challenges, LPE continues to be the favored method over techniques such as CVD, PVD, solvo/hydrothermal, and mechanical exfoliation for the production of various 2D materials. This preference is underscored by the successful synthesis of widely utilized 2D materials including graphene,^90−92^ TMDs,^81,93,94^ hBN,^81,95,96^ and emerging materials like phosphorene,^97−100^ borophene,^101,102^ silicene,^103,104^ MXene,^105−108^ selenene,^109−111^ tellurene,^112−114^ antimonene,^115^ and bismuthene,^116−118^ using LPE. Again, the research direction in the data driven can be expanded to cover those monoelement 2D materials in the future. The key factors contributing to its preference include the controllable quality/quantity of as-exfoliated flakes, cost-effectiveness, and the production yield. These qualities position LPE as particularly well-suited for various electrochemical applications, especially in the context of large-scale industrial production. Among various 2D materials, this review will primarily concentrate on the liquid phase exfoliation of TMDs, given their diverse array and remarkable properties applicable to various applications. Additionally, a comprehension of the convolutions of LPE concerning TMDs could potentially provide insights into the LPE processes of other 2D materials, as many of them share similar fundamental LPE mechanisms.

In this review, the as-exfoliated TMD prepared via liquid phase exfoliation is then highlighted with the electrochemical applications including energy storage, electrocatalyst, and potential-based membrane for ionic sieving. This review delves into the particulars of this technique, proposes future advancements, and explores approaches to enhance the yield of nanosheets while maintaining their electrochemical properties. Furthermore, we will explore the applications of TMDs, with a specific focus on electrochemical uses. The objective of this review is to provide a comprehensive compilation of techniques for augmenting the yield of TMDs nanosheets through direct liquid-phase exfoliation, encompassing various parameters like solvents, surfactants, sonication/shearing duration, and intensity. Beyond influencing exfoliation yield, these factors can impact the structural and chemical properties of TMDs nanosheets, potentially affecting their utility in electrochemical applications. Moreover, the road to data-driven techniques is then described to further expand the research direction of 2D materials and its electrochemical applications. Therefore, this compilation is intended to function as a pivotal information hub for the extensive community of researchers in electrochemical aspects including experimental and machine learning.

## 2 Liquid Phase Exfoliation

### 2.1 Background of Liquid Phase Exfoliation

**Figure 2:**
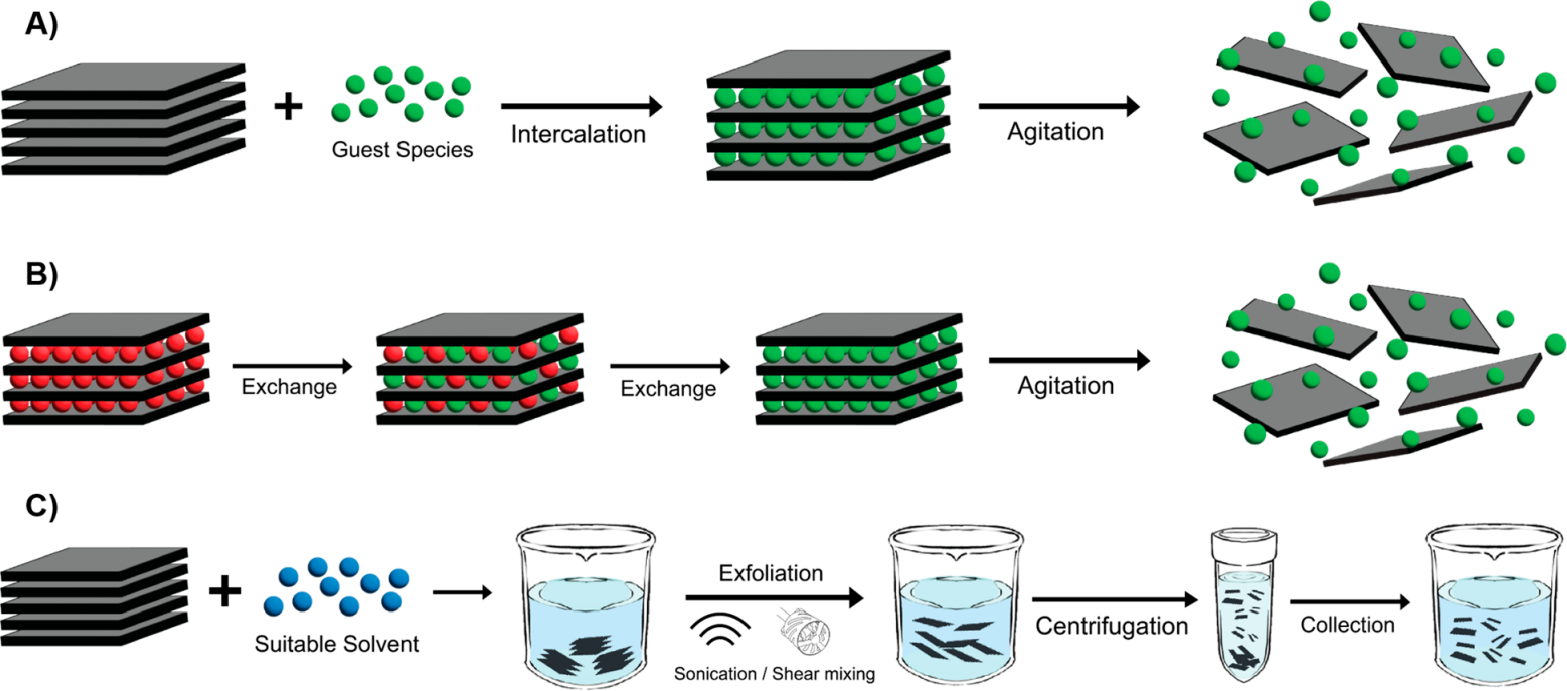
Schematic description of the main liquid phase exfoliation mechanism: (a) ion intercalation, (b) ion exchange, and (c) sonication/shearing-assisted exfoliation.

TMDs exist naturally in bulk, but for optimal utilization of their properties, it is preferable to have them in thin layers or monolayer nanosheets. This necessity arises from the thickness-dependent variations in the properties of TMDs. As bulk materials transition to mono- or fewer-layers, there is a transformation from an indirect to a direct band gap, leading to the emergence of distinctive mechanical, electrical, and optical characteristics in 2D TMDs.^93^ For instance, Splendiani et al. conducted a study where they calculated the band structures of bulk, quadrilayer, bilayer, and monolayer MoS_2_.^35^ They determined the number of layers using optical contrast microscopy coupled with AFM images. Their findings revealed that while bulk MoS_2_ possesses an indirect band gap of approximately 1 eV, monolayer MoS_2_ exhibits a modified energy band structure toward a direct electronic transition, with an energy bandgap of about 1.8 eV.^35^ This change can be attributed to an increase in the indirect band gap caused by significant quantum confinement effects in the out-of-plane direction as the material dimensions are reduced to few layers.^35,119^ This crucial shift from an indirect band gap presents numerous opportunities for exploring the undiscovered exceptional electronic properties of thin MoS_2_ films.^120^ Therefore, to commercialize 2D TMDs in real-world applications, cost-effective and scalable methods are crucial. Liquid phase exfoliation (LPE) is an attractive approach for producing solution-processed 2D materials, ideal for electrochemical applications requiring large quantities at a low cost. These materials were applied across various fields, including composites, energy storage, electrocatalysts, and electrochemical sensors. Moreover, they were utilized in ion-sieving membranes and electrochemical absorption systems. As depicted in Figure 2, the primary techniques for liquid exfoliation encompass ion intercalation, ion exchange, and sonication/shearing-assisted exfoliation.^83^

When employing lithium ions as the intercalant in both ion intercalation and ion-exchange methods, it becomes possible to attain a substantial monolayer yield of TMDs nanosheets (>90%). By controlling the applied potential to mobile ions, it can also alter the polymorph of TMDs materials. Ejigu et al. show that the electrochemical exfoliation (intercalation) using lithium perchlorate salt can turn hexagonal MoS_2_ (2H-phase) to a trimetallic MoS_2_ (1T-phase).^121^ This results in exceptionally high film quality and meets the requirements for satisfactory electrical performance in thin films.^122−125^ Numerous studies have explored the use of various ions, including different alkali metals, magnesium, zinc, and ammonium ions, for both chemical and electrochemical intercalation. Among these options, lithium-ion intercalation has proven to be the most efficient.^126−128^ However, this situation poses a considerable challenge, given that the flammability of the lithium-ion source introduces substantial hazards.^120^ This concern extends beyond laboratory environments to industrial scales, making it impractical for upscaling. Another limitation of this technique is that the phase transitions from the 2H to the 1T phase after exfoliation due to the thermodynamic stability of the 2H phase.^121,129^ This transition occurs when an excess charge is introduced, altering the unit cell structure from trigonal prismatic to octahedral.

On the other hand, sonication/shearing-assisted liquid phase exfoliation (a so-called solvent-assisted liquid phase exfoliation) stands out as a gentle and scalable method that is not affected by moisture and temperature.^130^ The exfoliation process comprises the following steps: (i) bulk TMDs are submerged in a solvent (which may consist of pure solvent, a combination of solvents, or a solution containing a dispersant), (ii) ultrasound or shearing assistance with a controlled power is then applied to exfoliate the bulk TMDs, and (iii) the resulting supernatant, containing finely dispersed and thin nanosheets, is separated by centrifugal techniques from any unexfoliated materials in the dispersion (see Figure 2c). In fact, the effectiveness of the exfoliation process ultimately determines the flake properties and the yield of TMDs nanosheets. Again, a variety of the as-obtained flake properties play a significant role in the electrochemistry of those materials. Thus, it is important to understand the preparation of the samples; hence, it can create a linkage between materials synthesis and electrochemical performance. Considering factors such as sonication, the quantity/type of dispersant or surfactant used, as well as centrifugation speed and duration is key to the exfoliation. In this review, the influences of the solvent, encompassing dispersants/surfactants and mixed solvents are examined. Furthermore, the impact of other factors such as exfoliation power and duration, centrifugal speed, and the initial concentration of materials on the efficiency of exfoliation is subsequently discussed. Our prime goal is to consolidate all of these given parameters and define the optimized conditions necessary to achieve efficient liquid phase exfoliation of TMDs nanosheets. Throughout this process, the cost-effectiveness and environmentally friendly synthesis techniques were also explored for further use in electrochemical applications, e.g., energy storage, electrocatalyst, ions adsorption, and selective via 2D TMDs membranes.

### 2.2 Ultrasonication-Assisted Liquid Phase Exfoliation

**Figure 3:**
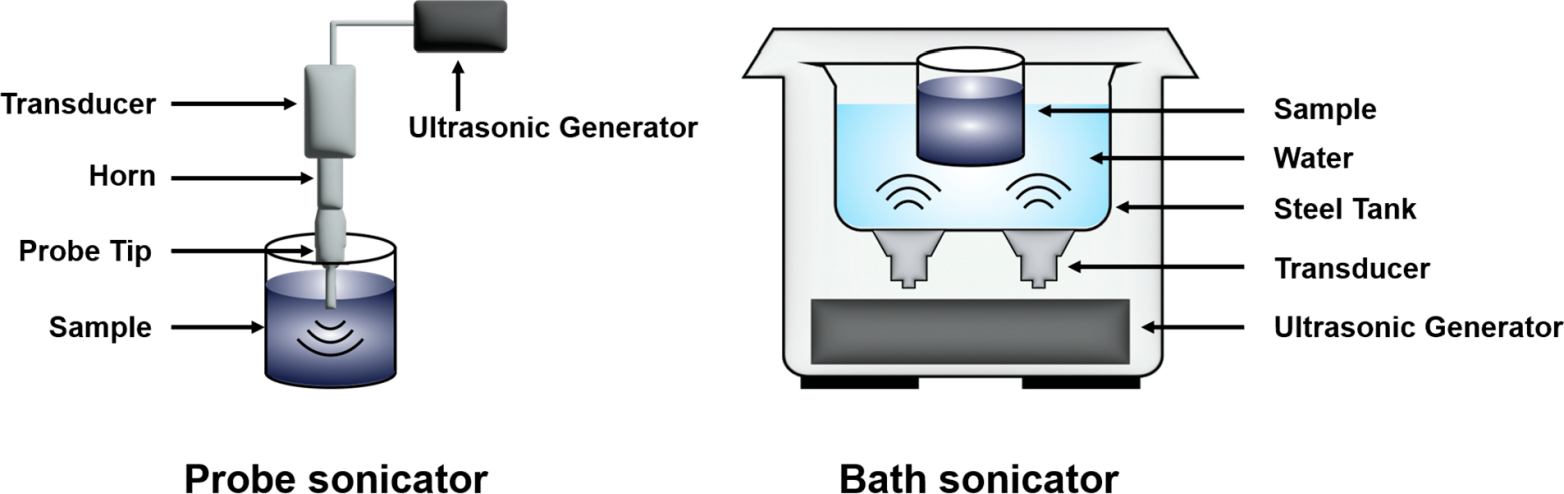
Schematic representation of probe sonicator and bath sonicator.

In the ultrasonication-assisted liquid phase exfoliation process, the introduction of ultrasonic waves to the bulk material leads to the formation of cavitation bubbles with significant surface energy and kinetic energy within the liquid.^131^ Cavitation is the phenomenon where micron-sized bubbles or voids in the liquid grow and then rapidly collapse due to pressure fluctuations.^132,133^ This cavitation effect acts on the bulk material and promotes exfoliation, reducing it into fewer nanosheets.^134,135^ The sonication parameters that can impact the yield and properties of nanosheets include the type of sonication, as well as the duration and intensity of sonication. There are two types of sonication: direct and indirect sonication. Direct sonication can be performed using a probe sonicator, while the latter is achieved with a bath sonicator. Following the dispersion of bulk powder in a suitable solvent, nanosheets are exfoliated using a sonicator probe, a sonicator bath, or a combination of both methods. Both methods utilize ultrasound for material processing, but their mechanisms, efficiency, and processing capabilities differ significantly. Figure 3 illustrates the probe sonication and bath sonication. Typically, the exfoliation process involves about 5 to 7 h of probe sonication, with 60% of the total power (ranging from 100 to 300 W) delivered in cycles of 6 s on and 2 s off.^87,136^ Alternatively, it may take 10 to 20 h of bath sonication, with an operative power-to-tank capacity ratio of 10 to 20 W L^–1^, for a 50 to 100 mL dispersion starting with an initial concentration of 20 to 50 mg mL^–1^.^91,137^ In probe sonication, the high-energy signals produced by the tip are directly applied to the dispersion media. Conversely, in an ultrasonic bath sonicator, there is no direct contact between the probe and the sample. Instead, the ultrasonic energy is transmitted from the horn to various containers containing the sample through the surrounding water medium.

**Figure 4:**
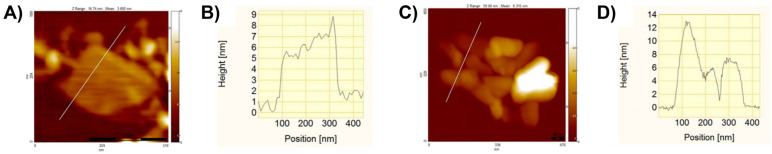
Impact of sonication power on the thickness of as-exfoliated TMDs nanosheets. AFM images of MoS_2_ after ultrasonic treatment with (a) 350 W and (c) 550 W; the images (b) and (d) display the height profiles along the white lines shown in (a) and (c), respectively. Reprinted with permission from ref (142). Copyright 2014 Elsevier.

The probe sonicators have been found to produce significantly higher concentrations of graphene dispersions within a relatively shorter time (reaching 2 mg mL^–1^ after 6 h)^138^ compared to ultrasonic baths (reaching 0.6 mg mL^–1^ after 100 h). However, it is worth noting that the graphene sheets in the probe dispersions are generally smaller compared to those obtained through bath sonication. This suggests that an increase in power likely aids in breaking down nanosheets,^138^ and similar findings have been observed in the exfoliation of MoS_2_ and WS_2_.^39,139,140^ The choice of acoustic power input depends on the desired characteristics of the TMDs nanosheets dispersion required for specific applications. For example, a study conducted by Qiao et al. found that there is a nonlinear relationship between the lateral dimensions (on average) and the concentration of nanosheets with varying ultrasonic powers during MoS_2_ dispersion.^141^ The optimal power range for their system was found to be between 250 to 320 W, as both lower and higher power settings had trade-offs in terms of exfoliation outcomes. Lower power yielded less exfoliated energy as it inhibited cavitation, while higher power (above 320 W) led to a higher concentration of cavitation bubbles, which in turn shielded the nanosheets from these forces efficiently. Additionally, lower power settings resulted in larger flakes and thicker sheets. Similar studies conducted by Zhang et al. have shown that increased power enhances the concentration of the exfoliated material, while simultaneously reducing the thickness and lateral size of the dispersed sheets,^142^ as shown in Figure 4a–d. However, the probe tip sonicator generates greater heat than the bath-type sonicator despite having substantially higher power than the latter, which may increase the danger of some solvents degrading. The total encapsulation is more remarkable when utilizing the bath-type sonicator in contrast with the probe-tip sonicator, and the management of heat production is significantly more straightforward in the bath-type sonicator than the probe-tip sonicator.^143^ Additionally, the most popular method for high-volume preparations is bath sonication.^144^ According to Han et al., the probe sonicator’s high-speed jets and powerful shock waves might reduce the size of the nanosheets or cause surface flaws, lessening their electrical and other valuable properties qualities.^145^ On the other hand, employing an ultrasonic bath sonicator results in fewer defects and causes less disturbance to the morphology and structure of the sheet. Furthermore, taking into account the solvent’s surface tension and solubility properties, extremely reactive radicals and byproducts produced by the sonolysis of organic solvent molecules may unintentionally affect the dispersion of nanomaterials. The morphological and chemical features of the produced graphene oxide (GO) sheets were compared as a result of using either a water bath or probe sonication, according to C. Mellado et al.^146^ Their findings demonstrated that applying the probe sonicator straight to the samples caused abnormalities between the layers, increasing the rugosity and degree of stacking and deteriorating the morphological features of the GO sheets. Additionally, materials that had undergone probe sonication revealed a substantial decline in the number of functional groups accessible for additional chemical synthesis. Therefore, they concluded that bath ultrasonication is better to probe ultrasonication for obtaining GO sheets since it is less invasive, makes it simple to control the specimen temperature, and results in fewer defects and less harm to the sheet’s structure and morphology. Exfoliated GO and MoS_2_ sheets are similar since their precursors are naturally abundant in layered forms. GO sheets also have the same uses as MoS_2_ in the areas of electronics, optics, chemistry, and energy storage.^147^

The duration of sonication plays a significant role in determining the concentration and quality of 2D material nanosheets. Many attempts have been made to increase exfoliation yield by extending the sonication time, with some experiments lasting up to 500 h.^148^ O’Neill et al. examined the impact of sonication time on MoS_2_ production, allowing for durations of up to 200 h.^149^ However, such prolonged methods demand high energy input and result in significantly smaller nanosheets due to increased scissoring.^149,150^ Nevertheless, they managed to achieve a high dispersion concentration of 40 mg mL^–1^ with relatively larger flakes, approximately 700 nm in size, after consistent sonication for 48 to 64 h.^149^ Similar outcomes were achieved using ultrasonic baths.^81^ Despite these achievements, it is worth noting that Li and Zhu reported that excessive sonication could introduce defects in the 2D lattice structure and reduce the size of the flakes to a few thousand nanometers.^151^ This limitation can impact the utilization of 2D nanosheets in large-scale integrated circuits and electronic devices.^151^ Sonication also affects the temperature of the medium, which increases linearly with longer sonication times, serving as an indicator of the energy input.^145^ The duration of sonication does not just influence the morphology of nanosheets but also influences certain properties, such as their electrocatalytic capability. For instance, in the case of WS_2_ from various sources,^152^ longer sonication times led to poor performance in the hydrogen evolution reaction (HER). As mentioned earlier, the size of nanosheets decreases as sonication time is extended, resulting in smaller nanosheets with larger surface area and more edge sites, which could potentially offer better catalytic properties. However, in the case of the as-prepared WS_2_, the trend was the opposite. Numerous studies indicate that acceptable concentrations of TMDs nanosheets with good quality can be achieved within the typical range of sonication times. It is important to note that the reported dispersion concentration or exfoliation yield is measured after the centrifugation process. For example, Coleman’s group used probe sonication at 285 W for 1 h in NMP, obtaining an exfoliation yield of 0.3 mg mL^–1^ for MoS_2_ and 0.15 mg mL^–1^ for WS_2_, with nanosheets retaining their normal structural and chemical properties.^81^ Additionally, using a horn probe sonic tip for 3 h in a 50% v/v aqueous ammonia solution,^153^ they achieved dispersion concentrations of 0.5 mg mL^–1^ for MoS_2_ and 1 mg mL^–1^ for WS_2_. Similar results were obtained using a bath sonicator, where they achieved an exfoliation yield of 1.26 mg mL^–1^ for MoS_2_ with excellent electrochemical adsorption properties after sonication in an ultrasonication bath for 12 h, using a water and NMP mixture as a solvent.^34^ Furthermore, with the use of surfactants, sonication times of 5 h in an ultrasonication bath led to concentrations of around 12 mg mL^–1^ for MoS_2_ nanosheets when using P123 as a surfactant and around 3 mg mL^–1^ for WS_2_ nanosheets with DBDM as a surfactant.^154^ It is important to note that these concentrations were achieved with relatively high surfactant concentrations of 12 mg mL^–1^ and 10 mg mL^–1^ for P123 and DBDM, respectively. While shorter sonication times may reduce costs and energy consumption, it is essential to consider the cost and energy required for surfactant removal from the final nanosheets and the cost of the surfactants themselves. It is crucial to note that exfoliation efficiency depends on multiple factors, including the solvent, surfactants, and centrifugation processes, in addition to sonication time.

### 2.3 Principal of Solvent-Selection for Liquid Phase Exfoliation

The TMDs nanosheets can be produced by placing bulk materials into various solvents and applying ultrasound treatment. However, the choice of solvent is a critical factor given the abundance of options available. In general, an appropriate solvent must satisfy the following criteria:^81,83,137,149,155,156^ (i) it should possess the capability to efficiently exfoliate the material at the highest feasible concentration and (ii) it must have the ability to stabilize the exfoliated 2D materials for an extended period. However, there is no precise recipe that describes the interactions between solvents and TMDs, which makes it challenging to delve into the mechanism of exfoliation processes. As a result, the majority of research efforts are concentrated on examining the thermodynamics of solvents during the exfoliation process. It has been emphasized that to achieve an effective liquid phase exfoliation and a high-yield synthesis. The Gibbs free energy of mixing (Δ*G*_mix_) should be minimized when the solvents are mixed with bulk TMDs.^136^ The free energy of mixing is calculated as follows:
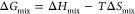
1where Δ*H*_mix_ represents the enthalpy of mixing, Δ*S*_mix_ denotes the entropy of mixing, and *T* is the absolute temperature of the surroundings. In the context of typical molecular solutes, a significant entropy of mixing can often drive a spontaneous mixing process (indicated by a negative Δ*G*_mix_), leading to the formation of what is commonly referred to as a solution. However, when handling 2D crystals with sizes spanning from several tens of nanometers to microns, the Δ*S*_mix_ is insufficient to spontaneously drive the process by surpassing the Δ*H*_mix_ and yielding a negative Δ*G*_mix_ for a spontaneous reaction. Therefore, it becomes crucial to minimize Δ*H*_mix_ through meticulous solvent selection.^157,158^ This step is essential to ensure the stable dispersion of exfoliated TMDs nanosheets in the solvent and also prevent restacking. The most straightforward way to describe the exfoliation and stabilization mechanism is to minimize the energy required to overcome the van der Waals forces between adjacent layers, with liquid immersion being the most promising and effective approach.^90^ It is evident that interfacial tension (surface tension) plays a crucial role when a solid surface is submerged in a liquid medium.^159^ Achieving a higher exfoliation yield is possible when the surface tension of the solvent closely matches that of the solute.^157,159−161^ This alignment in surface tension results in the reduction of Δ*H*_mix_. The correlation between surface tension and Δ*H*_mix_ can be expressed by the following equation:
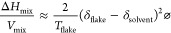
2where *T*_flake_ is the thickness of the flake of a layered material, ⌀ is the material volume fraction, and  is the square root of the component surface energy. Note that the surface energies of the liquids are approximately 30 mJ m^–2^ greater than their surface tension values.^162^ Coleman et al. successfully demonstrated that the graphene with high yield and high stability are obtained in organic solvents like *N*-methyl-pyrrolidone (NMP) and dimethylformamide (DMF)^163^ due to comparable surface energy. For example, these solvents have a surface tension close to 40 mJ m^–^^2^, which is roughly equivalent to surface energies of around 70 mJ m^–^^2^.^164^ Thus, it exhibited favorable interactions with graphene, which has a surface energy of approximately 68 mJ m^–^^2^.^163^ These concepts were then extended to various 2D materials, including BN, MoS_2_, and WS_2_.^81,87,149,165^ However, it is important to note that this phenomenon does not apply universally. The challenge arises from the fact that some solvents may possess seemingly appropriate surface tension values but exhibit low dispersed concentration. The solvents with similar surface tension values can vary significantly in their exfoliation capabilities.^82,166^ This issue is a prevalent challenge observed not only with graphene but also with transition metal dichalcogenides.^81,136,167^ Therefore, the surface tension matched can be applied for only a simplified approach, selecting an initial solvent.^81^ The widely recognized Hildebrand solubility parameter, *δ*_*T*_, has been employed for refining solvent selection. The enthalpy of mixing in a dispersion can be described by the equation:
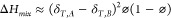
3where *δ*_*T,A*_ and *δ*_*T,B*_ represent the Hildebrand solubility parameters of the solute and solvent, respectively, while ⌀ signifies the solute’s volume fraction. Clearly, the dispersion is more favorable when the solubility parameters of the solvent and solute are in alignment. Again, a recurring challenge persists, where numerous solvents with similar Hildebrand solubility parameters demonstrate notably different exfoliation capabilities.^81,168^ This discrepancy arises because the Hildebrand parameter encompasses a comprehensive spectrum of solute–solvent interactions. Specifically, the Hildebrand solubility parameter, *δ*_*T*_, is derived as the square root of the cohesive energy density, *E*_C*,*T_/*V*, where *E*_C*,*T_ represents the total molar cohesive energy and *V* denotes the molar volume of the solvent. The Hildebrand parameter is typically well-suited for nonpolar systems. To achieve more precise solvent selection, the Hansen solubility parameters (HSP) are utilized. These parameters consider specific types of solute–solvent interactions, typically categorized into dispersion, polar, and H-bonding interactions, denoted as D, P, and H, respectively. The Hansen solubility parameters, represented as *δ*_D_, *δ*_P_, and *δ*_H_, are derived as the square root of the cohesive energy density specific to each component. Consequently, the square of the Hildebrand solubility parameter is equal to the sum of the squares of each Hansen solubility parameter:^81,82,157^
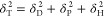
4

By integrating eq 3 and eq 4, the resulting enthalpy of mixing demonstrates that when all three solubility parameters of the solvent closely align with those of the 2D materials, it leads to the minimization of energy required for dispersion.^157,158^ Through the exfoliation of graphene in 40 different solvents, it is evident that solvents characterized by precise Hansen solubility parameter values lead to enhanced exfoliation and stabilization.^91,169−171^ The investigation was broadened to encompass 2D materials extending beyond graphene, including h-BN, MoS_2_, and WS_2_. In all cases, there is a distinct and pronounced peak observed in the Hansen parameters plot versus concentration.^81^ This highlights the necessity of optimizing all interaction energies for successful exfoliation and dispersion stabilization. Building on this approach, the researcher successfully obtained nanosheet solutions with high concentrations reaching up to 0.3 mg mL^–1^ for MoS_2_ and 0.15 mg mL^–1^ for WS_2_ in NMP.^81^ Hansen solubility parameters (HSP) are also applicable in the context of mixed solvents, as demonstrated by Zhou et al.^170^ The HSP, a semiempirical correlation featuring three parameters, is employed to elucidate dissolution behavior. The HSP distance *R*_*a*_, defined as eq 5, is used to characterize the dispersion of the solute.
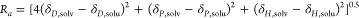
5

**Figure 5:**
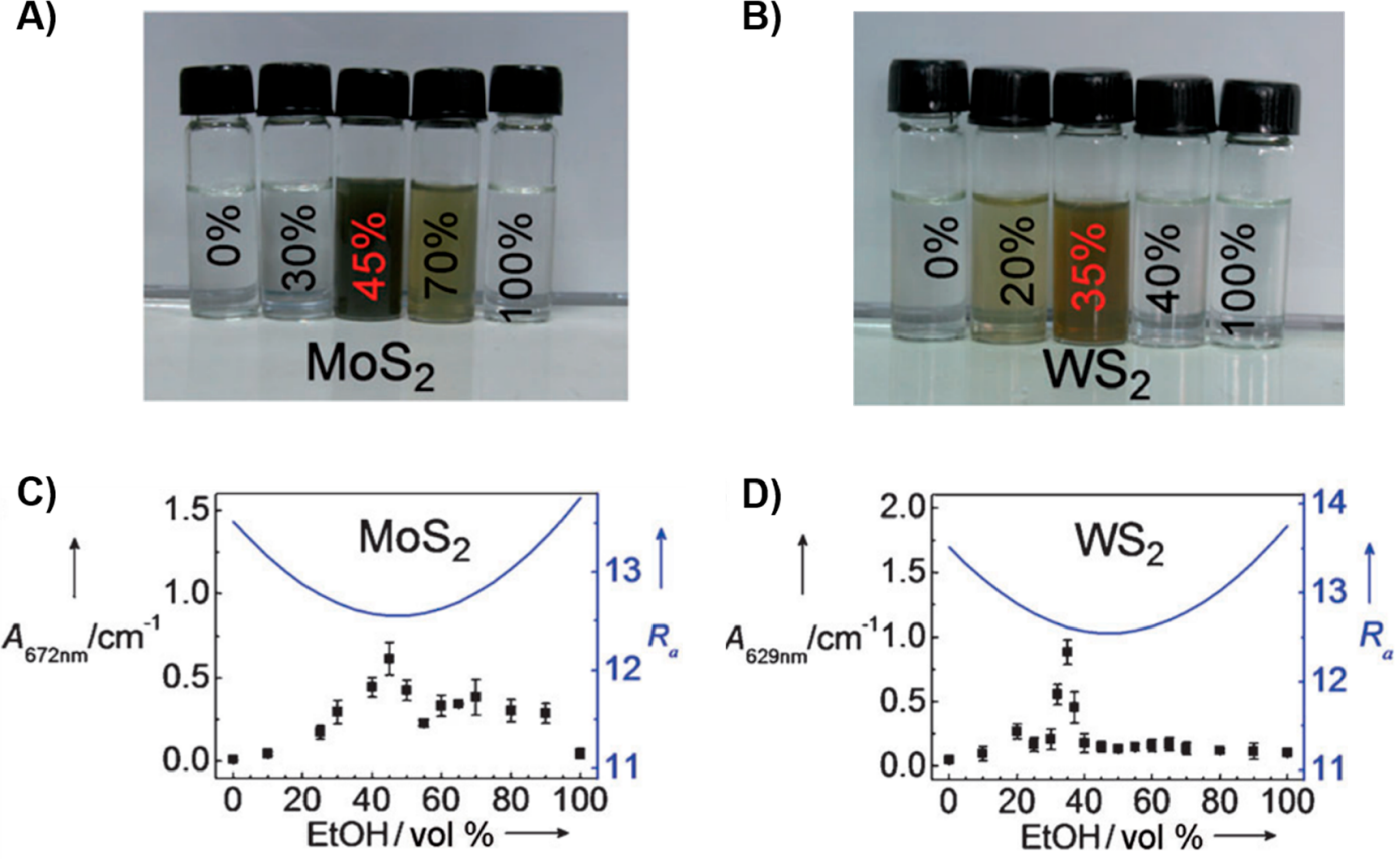
Photographs of (a) MoS_2_ and (b) WS_2_ dispersions in various ethanol–water mixtures, which have been stored under ambient conditions for a week. The absorbance of the (c) MoS_2_ and (d) WS_2_ suspensions in ethanol–water mixtures with different compositions are represented as dots, and the calculated *R*_*a*_ are shown as solid lines. Reprinted with permission from ref (170). Copyright 2011 Wiley.

A smaller *R*_*a*_ corresponds to a higher anticipated solubility. Moreover, this theory can be extended to solvent mixtures, as indicated by eq 6:
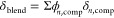
6where ϕ represents the volume fraction for each component. Consequently, this equation allows for the estimation of the mixture’s dissolving capacity for a nanomaterial. In light of these equations, Zhou et al. applied them to design mixed solvents for the liquid exfoliation of TMDs.^170^ They did so because they considered organic solvents like NMP and DMF to be toxic, with high boiling points and low volatility, making it challenging to remove these solvents from the final nanosheet product. To validate the concept that the solubility parameters in a mixed solvent are linked to both the volume fraction and the solubility parameters of each individual solvent, they experimented with a mixture of ethanol and water. These solvents were chosen as they are considered environmentally friendly, but individually unsuitable for the liquid exfoliation of TMDs materials. In their experiments, the highest MoS_2_ concentration achieved was approximately 0.018 mg mL^–1^ with a 45 vol % ethanol–water mixture, while for WS_2_, it reached about 0.032 mg mL^–1^ with a 35 vol % ethanol–water mixture. Figure 5a,b shows photographs of MoS_2_ and WS_2_ dispersions in various ethanol–water mixtures. The results underscore that the dispersion concentrations were highly influenced by the volume fraction of ethanol–water, and the concentration variation was contrary to the changes in *R*_*a*_ (see Figure 5c,d).

These results could imply the effectiveness of utilizing a mixture of green solvents based on Hansen solubility parameters. However, it is important to note that the dispersion yields achieved are still lower when compared to those prepared with organic solvents, which have surface tension values more closely matching those of TMDs nanosheets. This issue may arise from the limitation of Hansen solubility parameters, which may not fully capture the intricacies of interactions between solvents and 2D materials. The Hansen solubility parameters are fundamentally based on the assumption that solvents engage in only three distinct interactions, while other potential interactions are neglected. Consequently, this limitation can introduce uncertainty, making it challenging to predict the optimal exfoliation solvent with confidence. This is exemplified in the case where pyridine exhibits poorer exfoliation performance for graphene compared to NMP, despite pyridine having even closer Hansen solubility parameter values to that of graphene in comparison to NMP.^81^ Returning to the fundamental of thermodynamics, it is evident that efficient exfoliation requires a matching of the surface tension between the solvent and the solute. However, in practice, achieving this match can be challenging. The surface tension values of water, ethanol, and pyridine, for instance, do not align closely enough for achieving high exfoliation efficiency. The core of the issue lies in accurately determining the surface tensions of both the solvent and the layered materials, in addition to other solubility parameters, to make informed solvent selections. Measuring the surface tension of solvents is relatively straightforward, as it can be done with liquid/air tension. Nonetheless, when it comes to determining the surface tension of 2D materials, caution is essential. Surface tension measurements and calculations for 2D materials tend to exhibit significant variability, and calculations can lead to substantial overestimations.^162^ This is evidenced by the wide range of published data for materials like graphite and MoS_2_.^172,173^ To address this challenge, Shen et al. and Halim et al. introduced and applied Young’s equation.^91,155^ This equation links the interfacial tension between the solid and solvent with contact angles, offering a means to predict the appropriate solvent without relying on uncertain measurements or calculations of the surface tensions of layered materials. They both found that the use of a 7:3 IPA/water mixture as a solvent can achieve high production yield for MoS_2_ exfoliation. This solvent combination led to a notable increase in dispersion concentration when compared to other mixed solvents in their respective studies. Numerous studies reinforce the effectiveness of employing mixed solvents for the liquid exfoliation of TMDs. For instance, in the case of IPA/H_2_O exfoliation, the highest yield of WS_2_ nanosheets was achieved with a combination of 20 vol % isopropanol and 80 vol % water. This resulted in an exfoliation yield approximately 7 times higher than that achieved with pure IPA and 4 times higher than that achieved with pure water. This enhancement is attributed to the ideal alignment of the surface tension of WS_2_ with the binary solvent system.^174^ Adilbekova et al. explored an environmentally friendly approach to exfoliate MoS_2_ and WS_2_ by using a 50% v/v aqueous ammonia solution as a solvent, which proved to be superior to pure water and a greener alternative to organic solvents.^153^ They conducted the exfoliation process using a horn probe sonic tip, resulting in dispersion concentrations of approximately 0.5 mg mL^–1^ for MoS_2_ and 1 mg mL^–1^ for WS_2_. Other researchers employed a low-power exfoliation technique for WS_2_ using a binary mixture of acetone and water. They successfully obtained monolayers and a few layers with lateral dimensions in the nanometer range, achieving a yield of 2.7% with a solvent composition of 60% acetone in water.^175^ Esfandiari et al. describe an efficient approach to produce 2D WS_2_ sheets.^176^ The most elevated concentration of these nanosheets is attained through sonication in a mixture of water and dimethyl-sulfoxide (DMSO) with a DMSO-to-water ratio of 50%. This process yields individual monolayer and few-layer flakes with sizes in the range of about 1 μm. Dong et al. suggested that a mixed solvent of H_2_O_2_ and NMP can enhance the exfoliation yield of MoS_2_, achieving over 60 wt % under mild conditions.^177^ This improvement may be attributed to the spontaneous dissolution of MoS_2_ in H_2_O_2_. However, increasing the amount of H_2_O_2_ does not further enhance the exfoliation yield. Liquid-phase exfoliation of TMDs nanosheets requires careful solvent selection to minimize Δ*H*_mix_ by matching the surface tension of solvents closely with the solute, ensuring stabilization. However, achieving this alignment is challenging, leading to discrepancies in exfoliation capabilities among seemingly similar surface tension values. Hansen parameters, considering dispersion, polar, and H-bonding interactions, provide a refined approach, but predicting optimal solvents remains challenging. Experimental studies with mixed solvents, like IPA/water and H_2_O_2_/NMP, have demonstrated improved exfoliation yields for TMDs, emphasizing the potential of tailored solvent systems for efficient liquid-phase exfoliation.

**Figure 6:**
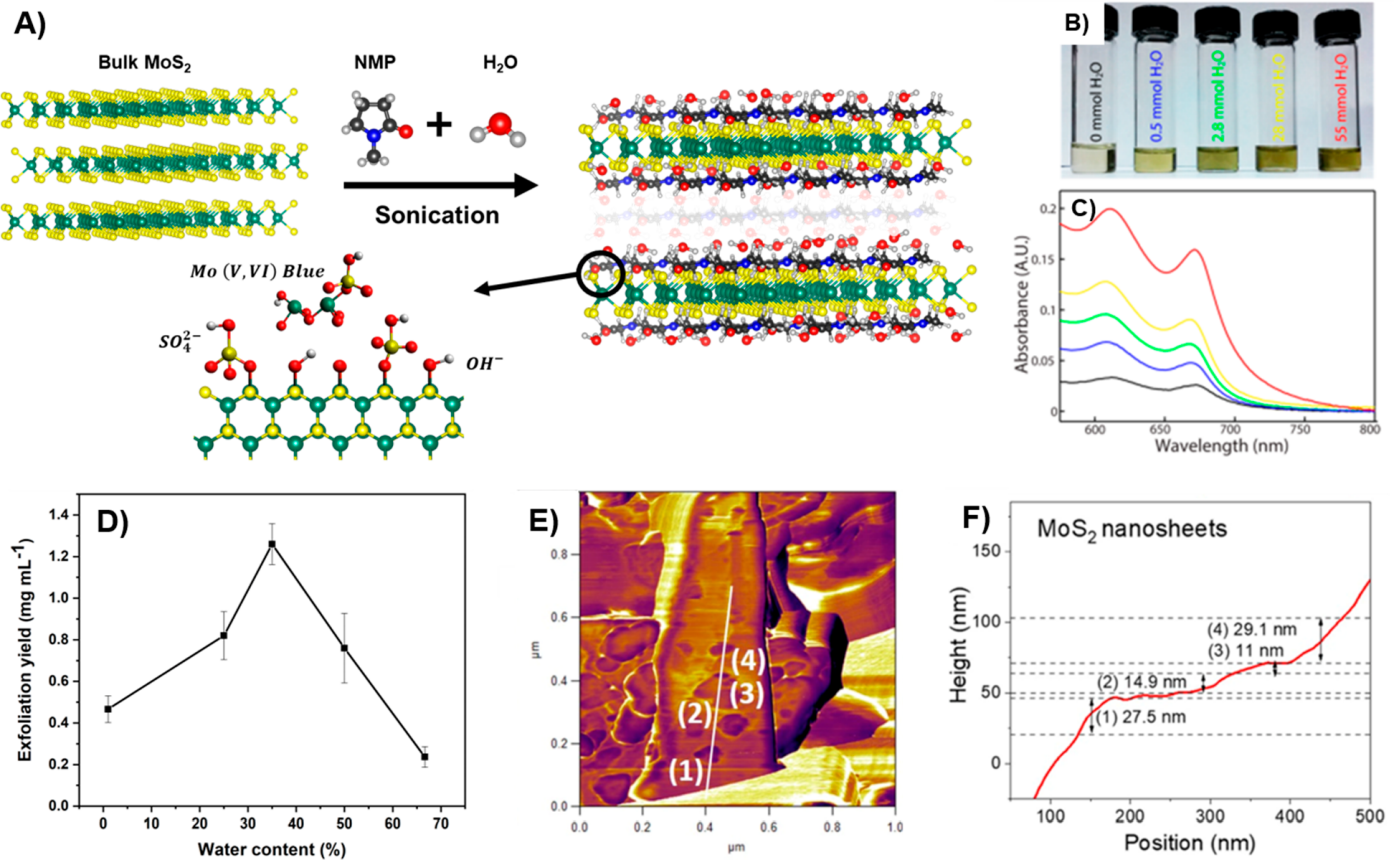
Impact of adding water on MoS_2_ exfoliation in NMP. (a) Schematic illustration of the mechanisms and chemical reactions involving NMP and water during the sonication of MoS_2_. (b) Visual representation of increased exfoliation of MoS_2_ flakes as varying amounts of water (0–55 mmol) are introduced to anhydrous NMP. (c) Corresponding absorption spectra of exfoliated MoS_2_ solutions obtained after 1 h of sonication and subsequent centrifugation at 12,700 rpm. Panels b,c: Reprinted from ref (93). Copyright 2016 American Chemical Society. (d) Exfoliation yield of MoS_2_ nanosheets prepared with different quantities of water in NMP. (e) AFM image of MoS_2_ nanosheets exfoliated in a 35% v/v mixture of DI water in NMP. (f) Height profile of the MoS_2_ nanosheets along the line scan shown in the panel. Panels d–f: Reprinted from ref (34). Copyright 2023 American Chemical Society.

In addition to mixed solvents, it is crucial to conduct experiments to gain insights into the mechanisms and chemical reactions of the solvent during sonication. *N*-methyl-2-pyrrolidone (NMP) is a commonly used solvent in liquid exfoliation, and direct exfoliation in NMP yields a dispersion concentration of approximately 7.5 mg mL^–1^ with lateral sizes of around 700 nm.^149^ To delve into the chemical reactions occurring during sonication in NMP, Jarwaid et al. revealed that the dissolved oxygen and moisture content in NMP could affect the exfoliation process (including structural and yield).^93^ They observed that higher moisture levels in NMP enhanced exfoliation efficiency (Figure 6b,c). This enhancement was attributed to the formation of redox-active species via autoxidation, which converted NMP to NMS through hydroperoxide intermediates. These reactive species facilitated exfoliation by oxidizing the edge sites of MoS_2_, thereby disrupting interlayer bonding and enabling electrostatic stabilization of particles in high-dipole solvents, see Figure 6a. Gupta et al. similarly demonstrated the importance of even trace amounts of water in NMP for improving exfoliation efficiency.^178^ They explained that this enhancement occurs because water localizes at the Mo-terminating edges of nanosheets, providing protection against erosion and enhancing the interaction between NMP and the nanosheets. Recognizing the potential benefits of these mechanisms, our group explored the liquid exfoliation of MoS_2_ using a significant amount of water instead of just trace amounts.^34^ We employed water as a cosolvent alongside NMP and fine-tuned the surface tension of this solution to closely match that of MoS_2_. Utilizing this method yielded a dispersion concentration of 1.26 mg mL^–1^, with a water volume of 35% in NMP, see Figure 6d. The AFM analysis (Figure 6e,f) revealed that the exfoliated MoS_2_ has a thickness of approximately 10–30 nm, indicating the presence of few-layered MoS_2_ nanosheets. Additionally, the lateral size of the exfoliated MoS_2_ falls within the range of 50 to 300 nm.

### 2.4 Surfactant-Assisted Liquid Phase Exfoliation

**Figure 7:**
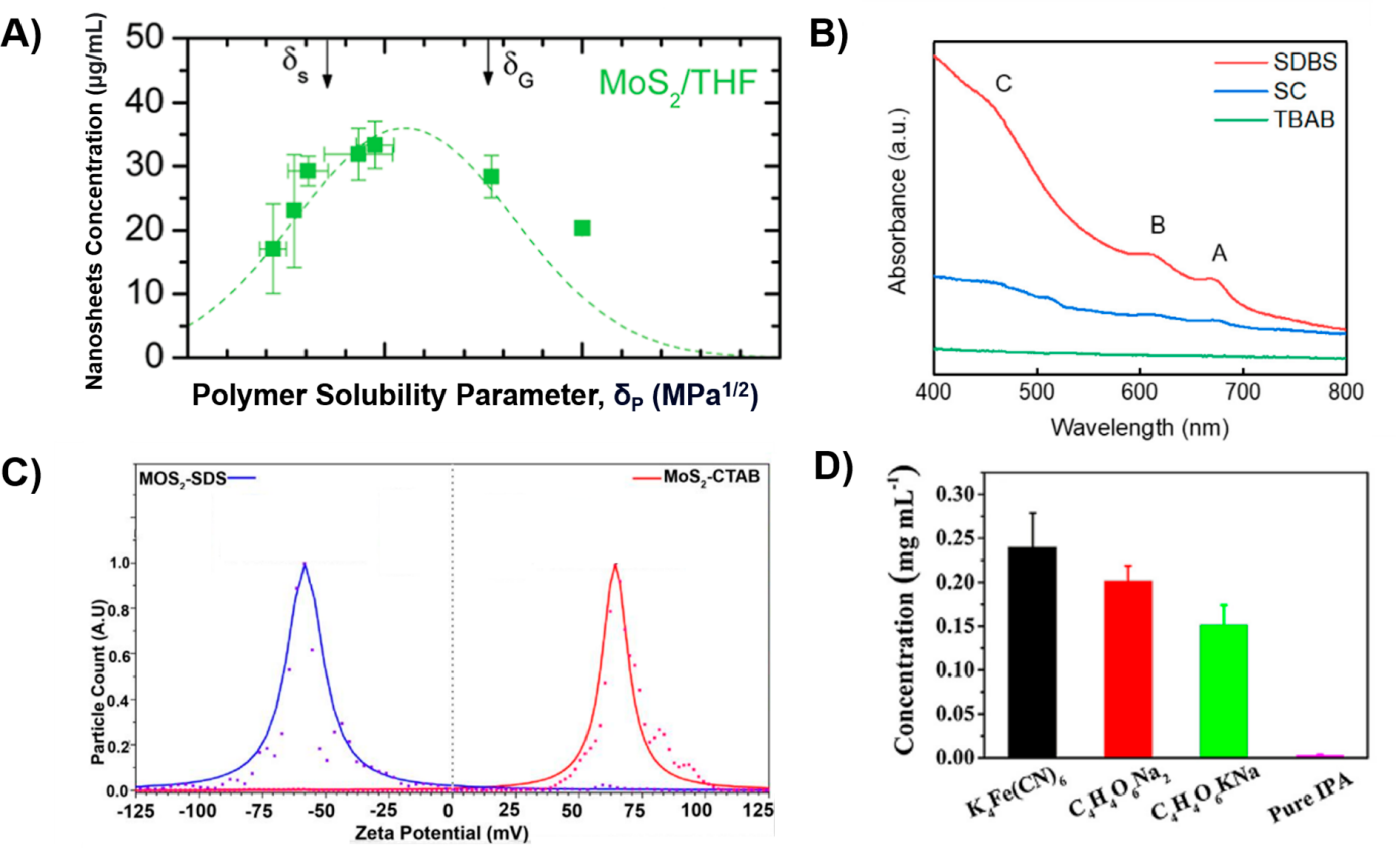
(a) The concentration of dispersed nanosheets, measured after centrifugation, plotted against the Hildebrand solubility parameter of the stabilizing polymer. Reprinted from ref (171). Copyright 2012 American Chemical Society. (b) UV–vis absorption spectra of solutions of MoS_2_ soaked in different surfactants (SDBS, SC, and TBAB) for 60 days. Reprinted with permission from ref (187). Copyright 2022 Elsevier. (c) Zeta potential distribution of the dispersions of exfoliated MoS_2_-CTAB and MoS_2_-SDS in water. Reprinted from ref (188). Copyright 2015 American Chemical Society. (d) Concentrations of the final MoS_2_ dispersions in pure IPA and IPA assisted with K_4_Fe(CN)_6_, C_4_H_4_O_6_Na_2_, and C_4_H_4_O_6_KNa. Reprinted with permission from ref (189). Copyright 2018 Elsevier.

While organic solvent-assisted is a straightforward and effective method for synthesizing 2D materials, it comes with limitations such as toxicity, environmental concerns, and high boiling points associated with these solvents. On the other hand, exfoliation in water and other greener, low-boiling-point solvents is less efficient and often leads to dispersions that reaggregate due to differences in surface tension. To overcome these challenges, many researchers have turned to additives and surfactants as stabilizers to enhance exfoliation efficiency. These surfactants/additives serve two main purposes: (i) adjusting the surface tension of the aqueous solution to better match that of the layered materials^179^ and adsorbing onto the surface of nanosheets leading to high yield production and (ii) creating additional repulsive forces that prevent aggregation through either electrostatic or steric mechanisms leading to high stable nanosheet dispersion.^136,180^ A wide range of additives can be employed for stabilization for TMDs nanosheets, including various types such as polymers,^181−184^ small molecules,^154,185,186^ ionic and nonionic surfactants,^136,187,188^ and inorganic salts.^189,190^ As an example, May et al. conducted research to explore the potential of polymer–solvent combinations with different Hildebrand solubility parameters for exfoliating and stabilizing layered nanomaterials.^171,183^ They experimented with nine different polymers in conjunction with tetrahydrofuran (THF) as a solvent. Their findings revealed that when these materials had closely matching solubility parameters, they could achieve the dispersion of exfoliated nanosheets. However, the resulting concentration of MoS_2_ nanosheets in the dispersion, shown in Figure 7a, fell within the range of 0.017–0.033 mg mL^–1^, which was relatively low. PVP was employed as a dispersant in water for the exfoliation of different 2D nanosheets using tip sonication.^184^ The highest resulting dispersion concentrations achieved were measured at 0.17 and 0.15 mg mL^–1^, with corresponding average lateral sizes of approximately 500 and 400 nm for MoS_2_ and WS_2_, respectively. Wang et al. harnessed pyrene-assisted liquid exfoliation for bulk MoS_2_ powder in ethanol, resulting in an exfoliated yield of 0.2 mg mL^–1^ for MoS_2_ nanosheets.^185^ Their study highlights that pyrene not only acts as an effective exfoliating agent, significantly expediting the exfoliation process, but also imparts exceptional solution dispersibility and stability to the resulting MoS_2_ nanosheets. In another investigation, it was revealed that a maximum concentration of about 3 mg mL^–1^ was attained for WS_2_.^154^ This achievement was made possible by employing n-dodecyl β-D-maltoside (DBDM), a small-molecule surfactant. It is worth highlighting that this necessitated a relatively high surfactant concentration of 10 mg mL^–1^. Smith et al. conducted TMDs exfoliation in water using probe sonication with the addition of the surfactant sodium cholate (SC).^136^ Their findings indicated that after 30 min of sonication, the concentration reached 0.048 mg mL^–1^, and this concentration increased to 0.5 mg mL^–1^ with an extended sonication time of 16 h. In terms of MoS_2_ yield, it amounted to approximately 10% when compared to the initial concentration. However, it is worth noting that MoS_2_ exfoliated in the presence of surfactants resulted in nanosheets with up to 10 stacked layers, indicating some reaggregation. Recently, Kaushik et al. introduced an innovative method for exfoliating substantial quantities of MoS_2_ through high-pressure liquid-phase exfoliation in deionized (DI) water,^187^ with the inclusion of surfactants like sodium dodecylbenzenesulfonate (SDBS), sodium cholate (SC), and tetra-*n*-butyl ammonium bromide (TBAB). Their findings highlighted that the use of SDBS led to highly efficient MoS_2_ exfoliation, surpassing the performance of the other two surfactants. The estimated yield achieved a remarkable 7.25%, and the nanosheet concentration reached 1.45 mg mL^–1^, marking one of the highest reported yields. The exfoliated MoS_2_ nanosheets, obtained from samples soaked for 60 days, consisted of 4 to 7 layers and exhibited stability in solvents for up to six months. The UV–visible absorption spectra of the solutions of MoS_2_ soaked in different surfactants SDBS, SC, and TBAB for 60 days are shown in Figure 7b. Furthermore, Gupta et al. stated that the use of surfactants has a notable impact on the surface charge of nanosheets.^188^ When employing cationic surfactant cetyltrimethylammonium bromide (CTAB), the nanosheets acquire a positive charge. In contrast, the use of anionic surfactants like sodium dodecyl sulfate (SDS) results in negatively charged nanosheets. The zeta potential (ζ) measurements of the MoS_2_ dispersions, prepared by sonication in the presence of CTAB and SDS, are depicted in Figure 7c. Zeta potential is a crucial parameter for assessing colloidal dispersion stability, indicating the surface charge magnitude and polarity within the double layer surrounding the colloidal particles. Typically, dispersions with zeta potentials more positive than +30 mV or more negative than −30 mV are considered stable due to electrostatic repulsion between particles. Interestingly, while the magnitude and distribution of the potential are similar in both dispersions, the signs are opposite. In the CTAB presence, the zeta potential is positive, whereas it is negative in the SDS presence. Liu et al. employed a salt-assisted approach in isopropanol (IPA) to exfoliate MoS_2_ nanosheets.^189^ They determined that potassium ferrocyanide (K_4_Fe(CN)_6_) was the most efficient salt, resulting in a MoS_2_ nanosheet dispersion with a high concentration of 0.240 mg mL^–1^. The findings are shown in Figure 7d. Researchers enhance liquid-phase exfoliation of TMDs nanosheets using surfactants and additives to match surface tensions. Various compounds, including polymers, small molecules, and surfactants, are explored. Innovative methods like high-pressure exfoliation with SDBS achieve high yields and stable dispersions. Surfactants influence nanosheet surface charge, impacting colloidal stability. Salt-assisted approaches, like using potassium ferrocyanide, efficiently exfoliate MoS_2_ nanosheets in isopropanol, achieving high concentrations.

Hu et al. mentioned in their review on dispersant-assisted liquid phase exfoliation that, typically, only a limited number of surfactants yielded MoS_2_ concentrations exceeding those achieved using mixed solvents or organic solvents, given similar experimental conditions.^96^ Furthermore, the inclusion of surfactants or additives to improve exfoliation has several drawbacks, such as increased costs and energy consumption for surfactant removal. Incomplete removal of surfactants can limit the usability of nanosheets for various applications. For instance, electronic applications often require a pure solvent to avoid introducing impurities into the exfoliated 2D materials. Even when 2D films are prepared using deposition techniques like drop-casting or spraying, they may still contain surfactants like SBDS, which can strongly adhere to 2D materials and are challenging to completely eliminate. Thermogravimetric analysis (TGA) suggests that the final 2D TMDs may retain approximately 1% of SDBS,^190^ which can adversely affect their electronic properties and hinder electronic applications. Certain additives, such as BSA, can be partially eliminated by subjecting the mixture to multiple high-speed centrifugation steps with water to harness centrifugal force. However, this process resulted in some reaggregation of the MoS_2_ nanosheets.^191^ Furthermore, the drawback of introducing polymers is also the challenge of removing them from the nanosheet surfaces postexfoliation. Nevertheless, it is important to note that the presence of polymers can also offer significant advantages. Polymers that adhere to the material surface following exfoliation provide opportunities for fine-tuning the physical properties of the ink, which can be advantageous for electronic printing applications.^192^ Similar to graphene, incorporating surfactant molecules can enhance the unique properties of TMDs nanosheets. For instance, coating WS_2_ nanosheets with a polymeric surfactant, PEO-PPO-PEO, preserved their luminescent properties by increasing interlayer spacing, keeping them electronically separate. As the aggregation of nanosheets was prevented, the stable nanosheet dispersion could be applied in various applications.^193^

### 2.5 Effects of Starting Concentration

**Figure 8:**
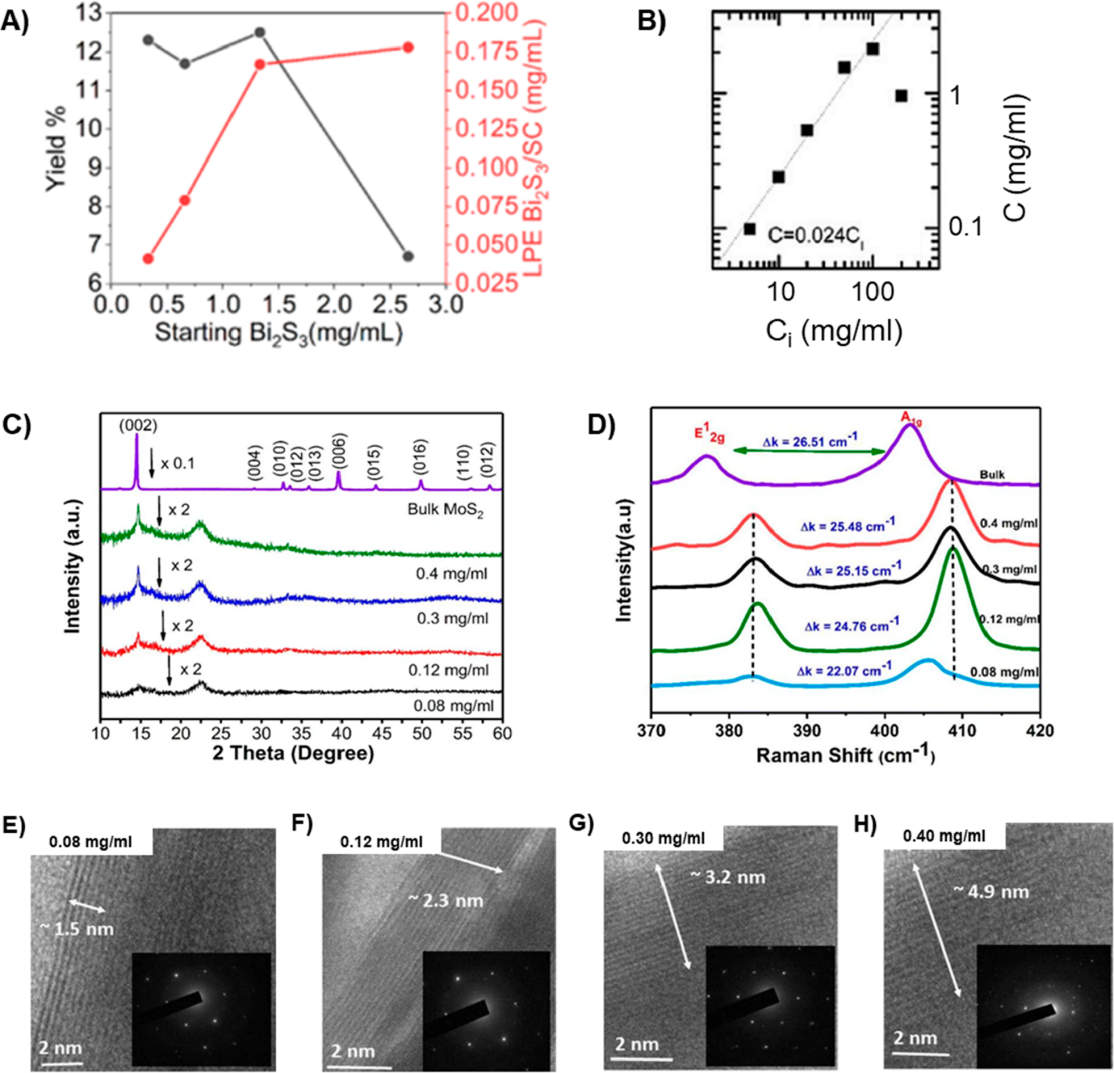
Effect of initial bulk concentration on the final TMDs nanosheets. (a) Exfoliation yield (black line) and concentration of LPE Bi_2_S_3_ in SC/H_2_O suspensions (red line) after 3 h of sonication at different initial bulk Bi_2_S_3_ concentrations. Reprinted under the terms of the CC-BY 4.0 license from ref (195). Copyright 2023 MDPI. (b) Concentration of dispersed MoS_2_ as a function of the initial MoS_2_ concentration. Reprinted with permission from ref (145). Copyright 2014 Springer Nature. (c) X-ray diffraction pattern comparisons between pristine MoS_2_ powder and nanosheets produced with different initial concentrations. (d) Raman spectra for bulk MoS_2_ and nanosheets from various initial bulk concentrations. HRTEM images of MoS_2_ nanosheets obtained from initial bulk concentrations of (e) 0.08, (f) 0.12, (g) 0.3, and (h) 0.4 mg mL^–1^. Figure 8c–h: Reprinted under the terms of the CC-BY 4.0 license from ref (120). Copyright 2020 Springer Nature.

In addition to the choice of solvent and surfactants, the concentrations also play a crucial role in exfoliation efficiency. There is an optimal range for the initial concentration of 2D materials in the solution. A low concentration hinders efficient exfoliation because there are not enough 2D material layers to exfoliate effectively.^187,194^ Conversely, overly high concentrations can lead to the clumping or restacking of the exfoliated layers, reducing exfoliation efficiency. Wang et al. conducted a study where they explored the impact of the initial bulk Bi_2_S_3_ concentration in surfactant-assisted LPE while keeping other factors constant.^195^ They observed that exfoliation yield remained consistently around 12% within the range from 0.33 mg mL^–1^ to 1.33 mg mL^–1^ of initial Bi_2_S_3_ concentration. In this range, the exfoliation yield increased linearly with rising concentrations. However, at a higher starting material concentration of 2.67 mg mL^–1^, there was a significant drop in the exfoliation yield to 6.7%, resulting in a final LPE Bi_2_S_3_ concentration of only 0.18 mg mL^–1^. These findings are depicted in Figure 8a. Thus, an increase in the initial bulk Bi_2_S_3_ concentration can boost the concentration of the final product, but this effect is limited, as the equilibrium of exfoliated nanosheets in the solution has been attained. Bodík et al. conducted experiments that revealed when the initial concentration of MoS_2_ exceeds a certain threshold, the exfoliation process undergoes significant oxidation, leading to the production of MoO_*x*_ nanoparticles in a water/ethanol solution during LPE.^194^ Their findings confirmed a direct relationship: the higher the initial MoS_2_ concentration, the greater the quantity of MoO_*x*_ nanoparticles generated. Within the initial concentration range from 1 to 10 mg mL^–1^, the predominant product consisted of exfoliated MoS_2_ flakes. However, they observed a decline in the yield of MoS_2_ flakes starting at an initial concentration of 5 mg mL^–1^. Once the initial concentration of MoS_2_ surpassed the 10 mg mL^–1^ threshold, the supernatant primarily contained MoO_*x*_ nanoparticles. Furthermore, according to Kaushik’s research, an increase in the initial MoS_2_ concentration could lead to a higher final concentration of nanosheets.^187^ This aligns with the findings from Coleman’s group, which indicated that the concentration of MoS_2_ nanosheets increases proportionally with the initial mass of MoS_2_ used and reaches its maximum when the initial MoS_2_ concentration is 100 mg mL^–1^ in NMP^149^ (Figure 8b). The research of Sahoo et al. demonstrated that, in contrast to the exfoliation yield, the quality of the exfoliated nanosheets decreased as the initial bulk concentration increased.^120^ They produced MoS_2_ nanosheets in various bulk initial concentrations of 0.08, 0.12, 0.3, and 0.4 mg mL^–1^ in acetone using ultrasonication. The weight of dispersed nanosheets was measured and calculated, with values of 1.13, 1.35, 1.58, and 2.07 mg for the respective concentrations. In the case of the 0.08 mg mL^–1^ initial concentration, XRD patterns, depicted in Figure 8c, showed a very low-intensity peak, indicating extremely thin nanosheets. Raman spectra revealed that nanosheets prepared with an initial concentration of 0.08 mg mL^–1^ exhibited the minimum frequency difference compared to the other concentrations, shown in Figure 8d. In addition, Figure 8e–h shows the transmission electron microscopy (TEM) images at low magnification and displays the height distribution of MoS_2_ nanosheets at various concentrations. The observed heights ranged from 1.5 to 4.9 nm, and it was evident that layer thickness increased with an increase in the MoS_2_ bulk initial concentration, consistent with XRD results. Furthermore, nanosheets prepared from 0.08 mg mL^–1^ showed the highest degradation of methylene blue dye at 45.6% when exposed to light. In comparison, samples prepared from 0.12 and 0.3 mg mL^–1^ showed 32.3% and 31.1% dye degradation, respectively, and this percentage gradually decreased to 27% for nanosheets synthesized from 0.4 mg mL^–1^. Thus, it can be concluded that the percentage of dye degradation decreased gradually with an increase in the number of layers, resulting from the increase in the initial bulk concentration.

**Figure 9:**
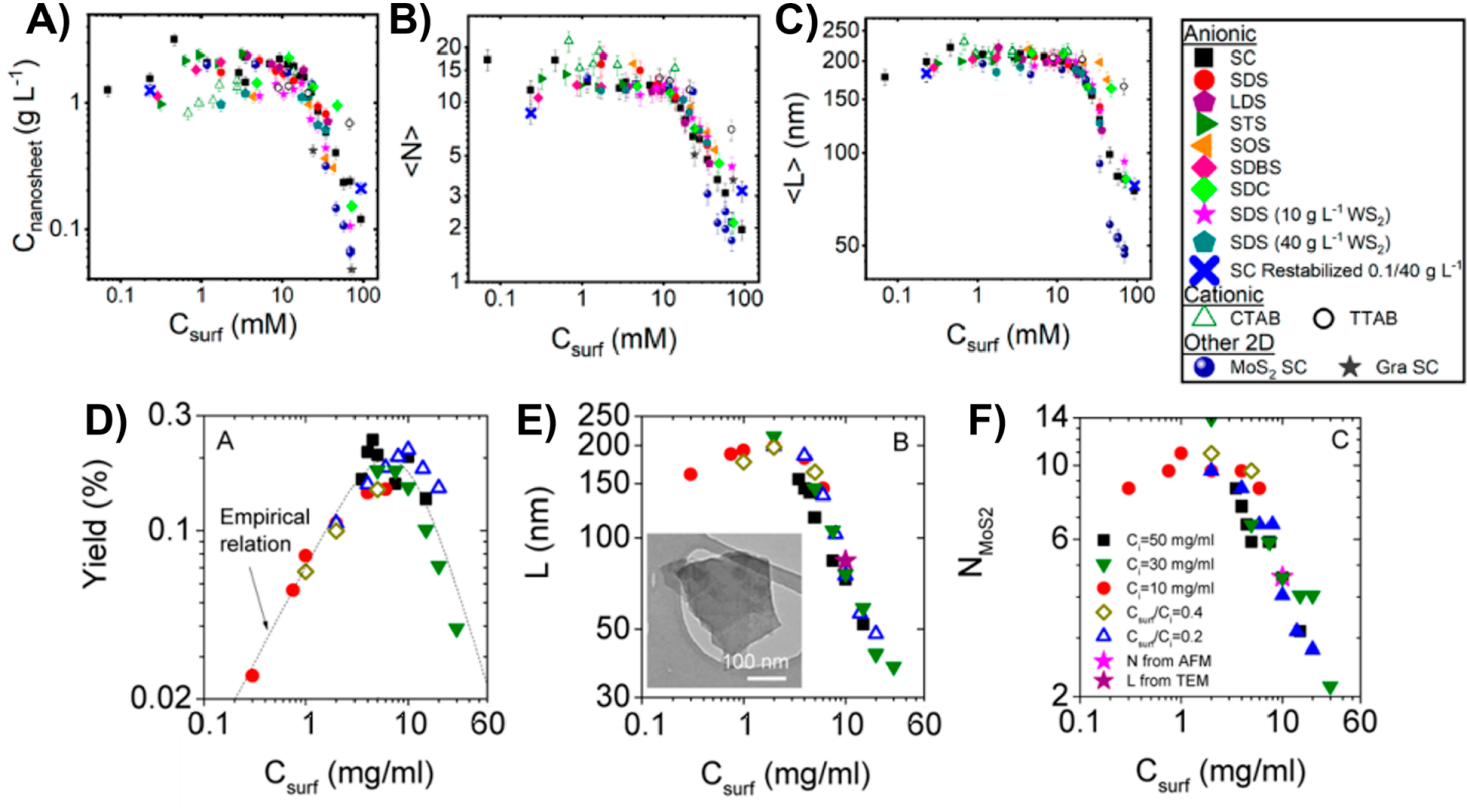
Effects of surfactant concentrations on exfoliated TMDs nanosheets. (a) Concentration of WS_2_ nanosheets in relation to surfactant concentration, expressed in mM, for various ionic surfactants. (b) Average number of layers ⟨N⟩ and (c) mean length ⟨L⟩ of nanosheets as a function of surfactant concentration. Figure 9a–c: Reprinted from ref (196). Copyright 2020 American Chemical Society. (d) Yield of MoS_2_ exfoliation, (e) lateral size ⟨L⟩ of nanosheets, and (f) thickness in terms of the number of layers ⟨N⟩ plotted against the concentration of aqueous SC. Figure 9d–f: Reprinted from ref (197). Copyright 2015 American Chemical Society.

Griffin et al. conducted a study to investigate how the concentration of surfactants affects the dimensions and yield of liquid phase exfoliation (LPE) of WS_2_.^196^ They performed a two-step sonication process, where the first step involved sonication in water, followed by centrifugation to remove the supernatant. The sediments were then redispersed in an aqueous solution containing various surfactants, including SC, SDC, SDS, STS, SOS, LDS, SDBS, CTAB, and TTAB. Their findings showed that, for ionic surfactants, the mass of nanosheets produced remained relatively constant at low surfactant concentrations but sharply decreased when surfactant concentrations exceeded around 10 mM, regardless of the surfactant used, depicted in Figure 9a. This trend was consistent for nanosheet length and thickness, with the average number of layers decreasing to as low as 2 and lengths as short as 50 nm at very high surfactant concentrations, illustrated in Figure 9b,c. Importantly, they found that only very small concentrations of surfactant (approximately 0.1 g L^–1^) were needed to maintain a stable dispersion. Overall, their results suggested that surfactant choice had less impact on nanosheet stabilization than one might anticipate. Varrla’s study involved adding a fixed concentration of bulk MoS_2_ to an aqueous solution prepared with various concentrations of SC and exfoliating it using a shear mixer.^197^ The study found that multiple processing parameters, including the initial MoS_2_ concentration, mixing time, liquid volume, and rotor speed, had an impact on both the concentration and production rate of nanosheets. The study achieved high concentrations of approximately 0.5 mg mL^–1^ and production rates of around 1 mg min^–1^. Interestingly, the lateral size and thickness of the nanosheets remained consistent with all the production parameters except for the surfactant concentration. By adjusting the surfactant concentration, the study demonstrated control over the lateral size, which ranged from approximately 40 to 200 nm, and the thickness, which could be varied between about 2 and 12 layers. The results indicated that as the surfactant concentration increased, the yield also increased, reaching a saturation point at surfactant concentration values between 5 and 10 mg mL^–1^. Beyond this point, the yield started to decrease significantly at higher surfactant concentrations. This observation aligned with the findings of Griffin’s work.^196^ The research of Varrla et al. emphasized a distinct relationship between the production yield, nanosheet thickness, and lateral dimensions, which were associated with the surfactant concentration denoted as *C*_surf_.^197^ The results are visually presented in Figure 9d–f. In Li’s research, it was observed that a higher initial concentration of bulk MoS_2_ did not result in a higher concentration of nanosheets.^198^ Interestingly, these findings differed from the conclusions reached in Varrla’s study.^197^ This disparity could likely be attributed to the use of distinct processing parameters and shear devices in the two studies.

### 2.6 Centrifugation-Based Size Selection

**Figure 10:**
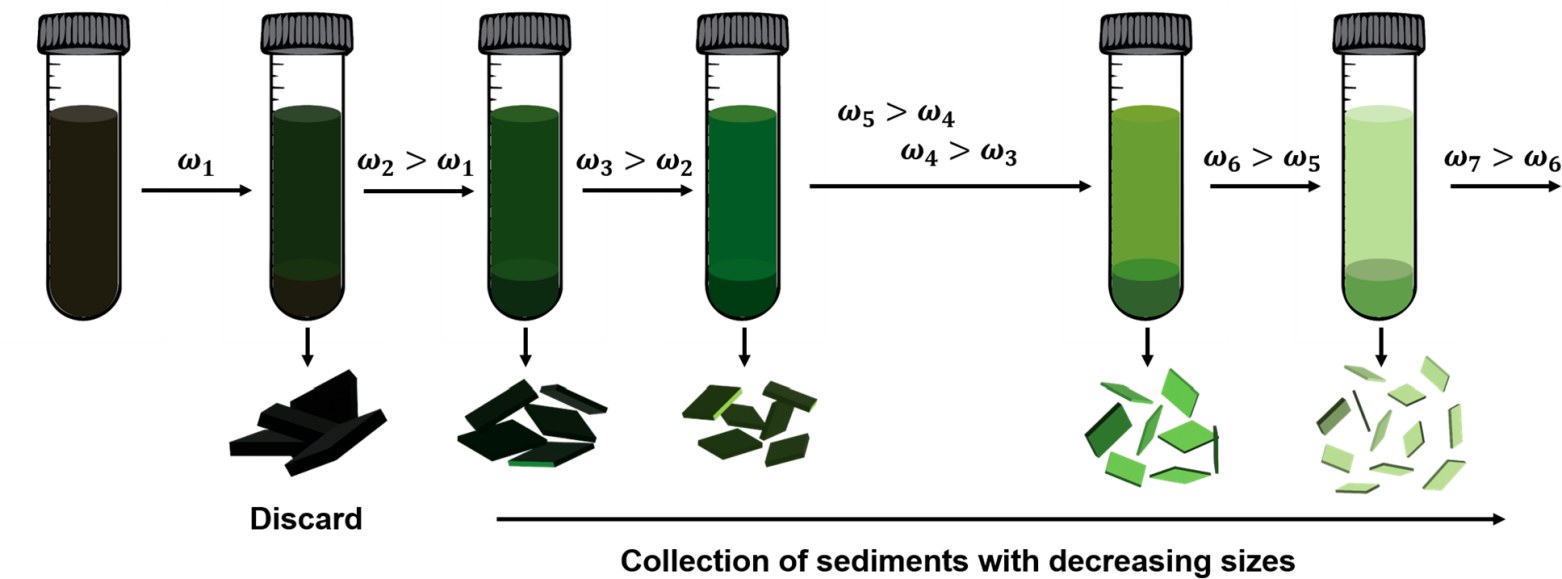
Schematic representation of liquid cascade centrifugation process. Nanosheets of specific sizes are gathered as sediments, with each sediment being captured or confined between two centrifugation speeds (ω). The process involves progressing from lower speeds to higher ones in sequential steps.

**Figure 11:**
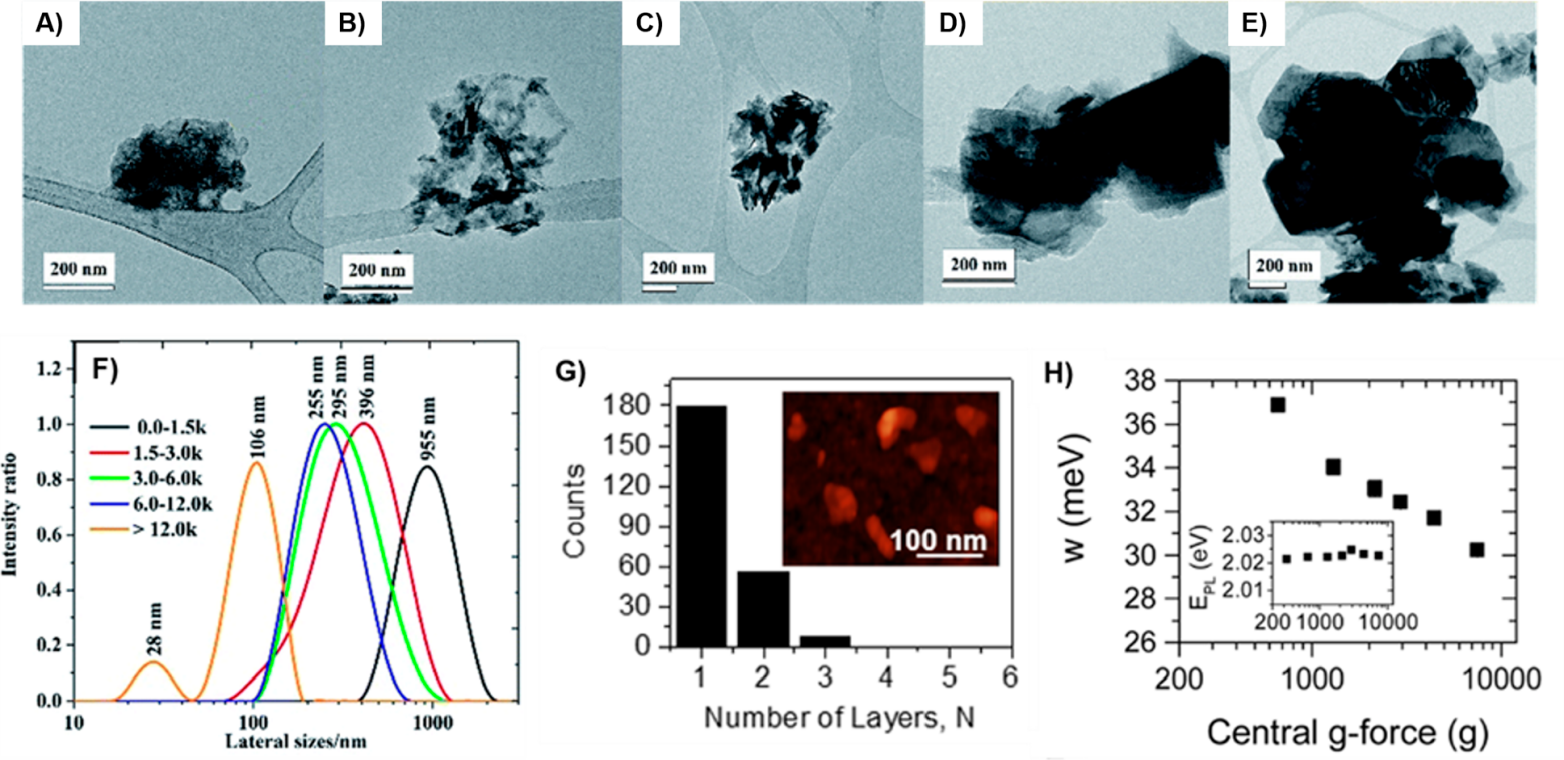
TEM images illustrate the size of WSe_2_ flakes at different centrifugal rates: (a) ≥ 12000 rpm, (b) 6000 to 12000 rpm, (c) 3000 to 6000 rpm, (d) 1500 to 3000 rpm, and (e) ≤ 1500 rpm. (f) WSe_2_ flake size distribution determined by dynamic light scattering (DLS). Figure 11a–f: Reprinted with permission from ref (28). Copyright 2021 Royal Society of Chemistry. (g) Histogram representing the thickness of WS_2_ nanosheets as determined by AFM, corresponding to the number of layers. It includes a representative image of WS_2_ nanosheets from a dispersion enriched in monolayers achieved through liquid cascade centrifugation. (h) Photoluminescence (PL) line width, plotted against central centrifugation acceleration, (g-force), and inset shows PL position as a function of g-force. Figure 11g–h. Reprinted from ref (199). Copyright 2016 American Chemical Society.

In liquid phase exfoliation, whether through ion intercalation, sonication-assisted exfoliation, or some other technique, e.g., shearing-assisted, the subsequent centrifugation step serves a dual purpose. Apart from eliminating unexfoliated materials, it also enables the selection of nanosheet sizes, which is a potentially crucial requirement for various applications. By harnessing the centrifugal force, where the technique is based on the weight of the nanosheets, centrifugation can effectively separate the 2D nanosheet size. The dimensions of TMDs flakes can be manipulated by adjusting the centrifuge speed. Many researchers have successfully employed liquid cascade centrifugation, proving it to be an efficient method for obtaining nanosheets of specific sizes.^28,199−202^ This process offers high yields, minimizes wastage, and can be tailored to produce nanosheets with desired dimensions (i.e., lateral length and thickness). Note that the cascade centrifugal is the most efficient way to control the lateral size of the as-exfoliated samples. Backes et al. explained that the mechanism behind this process, illustrated in Figure 10, entails a series of centrifugation steps, with each step having a higher speed than the previous one.^199^ After each step, the sediment retains nanosheets within a specific size range, effectively “trapping” them between two different centrifugation speeds. The lower speed step removes larger nanosheets into the previous sediment, while the higher speed step extracts smaller nanosheets into the supernatant. The sediment discarded after the initial centrifugation contains unexfoliated materials, whereas the supernatant discarded after the final centrifugation step contains extremely small nanosheets, implying that higher centrifugation speeds can produce thin and small flakes compared to lower speeds. This observation corresponds with the outcomes reported in our prior study,^28^ where the size of the WSe_2_ flakes was successfully controlled through centrifugation at speeds ranging from 1500 to 12000 rpm. Notably, at the highest centrifugal rate exceeding 12,000 rpm, the flake sizes measure less than 100 nm (refer to Figure 11a). On the other hand, when the centrifugal speed is reduced, the lateral dimensions of WSe_2_ flakes increase, falling within the range of 200 nm to 1 μm, as depicted in Figures 11b–e, respectively. These results exhibit a strong correlation with the lateral size determined via DLS analysis, presented in Figure 11f. Moreover, these findings are consistent with earlier reports on the exfoliation of graphene and MoS_2_.^200−202^ Moreover, employing the liquid cascade centrifugation method enables the collection of well-developed monolayer sheets. For instance, consider an aqueous dispersion of WS_2_ with an impressive monolayer content of up to 75%,^199^ as shown in Figure 11g. These monolayer-rich dispersions exhibit notably bright photoluminescence characteristics with narrow line widths (<35 meV), as demonstrated in Figure 11h. These features underscore the high quality of the nanosheets.^199^ In addition to liquid cascade centrifugation, increasing the exfoliation yield can be achieved through the recycling of sediment or unexfoliated bulk materials. For instance, Smith et al. recycled the sediments by redispersing them in the solvent and subjecting them to a second round of sonication.^136^ This process led to an overall increase in the yield of MoS_2_ nanosheets from 10% to 17%. However, it is important to note that recycling sediments can lead to a shift in flake distribution toward thinner flakes, potentially leading to a decrease in the quality of nanosheet such as the smaller lateral nanosheet size, more defect formations, as well as physicochemical properties.^82^

Besides liquid cascade centrifugation, nanosheet thickness or size-selection can be managed through density gradient ultracentrifugation (DGU). While liquid cascade centrifugation involves sequential increases in centrifugal force, DGU relies on a density gradient medium, typically a solution with a continuously increasing density gradient. At high centripetal acceleration, the material suspended in the dispersion is propelled to different points in the gradient due to differences in its buoyant density and that of the medium. Upon reaching its isopycnic point, where its buoyant density aligns with that of the medium, the material no longer experiences differential density-driven movement. Consequently, the material can be collected in fractions, taken layer-by-layer from the centrifuge tube.^203^ Green and Hersam demonstrated the application of DGU, aided by SC, for the solution-phase synthesis of graphene. In this method, exfoliated sheets were isolated with precise control over their thickness. They asserted that graphene dispersions created through density differentiation exhibit enhanced performance in transparent conductors compared to those generated through traditional sedimentation-based centrifugation methods. Their research indicates the potential for employing density differentiation to sort other two-dimensional nanomaterials as well.^203^

### 2.7 Overview of the Impact of Different Exfoliation Parameters on Exfoliation Efficiency

A thorough summary of the influence of exfoliation parameters, specifically, sonication time and power, surfactants, starting concentrations, and centrifugal speed, on the efficiency of TMDs exfoliation will be provided. This summary will encompass the yield and properties, including thickness and lateral dimensions, of the exfoliated nanosheets, taking into account both time and energy consumption. The initial exfoliation factor to be addressed is the sonication process, which includes parameters such as sonication power and duration. With regard to sonication power, increased power enhances the breakdown of nanosheets, facilitating higher exfoliation yield in less time. This process results in thinner and smaller flakes, albeit with more defects due to the elevated intensity. Conversely, lower power generates thicker and larger flakes due to reduced cavitation and exfoliation energy, consequently yielding lower quantities. Extending sonication time increases exfoliation yield but comes at a high energy cost, yielding thinner, smaller flakes with potential defects. Prolonged sonication not only impacts the morphology of the flakes but also influences their properties, such as exhibiting poorer HER performance. The subsequent parameter to be considered is the impact of surfactants. Surfactants serve the purpose of adjusting surface tension and creating a repulsive force to enhance exfoliation efficiency. However, there are drawbacks associated with the use of surfactants. One drawback is the escalation in the costs of the surfactants themselves, coupled with the increased costs and energy required for surfactant removal. Additionally, some surfactants cannot be entirely removed from the nanosheets, restricting their application in electronic contexts that demand high-purity TMDs. Surfactant concentrations also impact exfoliation efficiency. Increasing surfactant concentration initially enhances exfoliation yield, reaching saturation. Beyond this point, exfoliation yield decreases rapidly. This trend is consistent for nanosheet length and thickness: they increase until reaching an optimum point, after which they decrease. Another crucial parameter is the starting concentration of the TMDs, and its impact on exfoliation yield mirrors that of surfactant concentration. Increasing the starting concentration results in an initial rise in exfoliation yield, reaching saturation, and subsequently declining. This phenomenon occurs because excessively high concentrations lead to the clumping and restacking of dispersed nanosheets, potentially leading to the formation of oxide forms and altering their oxidation numbers. The final parameter to be addressed is the centrifugal speed. By adjusting the centrifugal speed, nanosheets with specific sizes can be obtained. Higher centrifugal speeds result in the production of thinner and smaller flakes compared to lower speeds. Additionally, varying the centrifugal speed allows for the collection of well-developed monolayer sheets. As an illustration, a specific dispersion achieved through liquid cascade centrifugation at the highest speed of ten thousand rpm has a monolayer content of up to 75%. To consolidate the impact of all previously discussed parameters, a comprehensive table was assembled to provide enhanced clarity (see Table 1).

**Table 1:** Impact of Different Exfoliation Factors on the Exfoliation Yield, Dimensions of Flakes, and Thickness in Transition Metal Dichalcogenides (TMDs)

Exfoliation parameters		Exfoliation yield	Flake dimensions	Flake thickness
Sonication duration^149^	Longer duration (at 140 h)	Higher (40 mg mL^–1^)	Small (≈ 0.2 μm in length)	Thinner
	Shorter duration (at 60 h)	Lower (≈ 8 mg mL^–1^)	Large (≈ 0.7 μm in length)	Thicker
Sonication power^142^	Higher intensity (at 550 W)	Higher (≈ 0.43 mg mL^–1^)	Small (≈ 100 nm in size)	Thinner (≈ 6 nm)
	Lower intensity (at 350 W)	Lower (≈ 0.36 mg mL^–1^)	Large (≈ 200 nm in size)	Thicker (≈ 12 nm)
Surfactant concentration^196^	Higher concentration (at C_surf_ of 30 g L^–1^)	Lower (≈ 0.25 g L^–1^)	Small (≈ 85 nm in length)	Thinner (2–3 layers)
	Lower concentration (at C_surf_ of 5 g L^–1^)	Higher (≈ 2.25 g L^–1^)	Large (≈ 200 nm in length)	Thicker (11–12 layers)
TMDs starting concentration^120^	Higher concentration (at C_i_ of 0.4 mg mL^–1^)	Lower (≈ 0.17% yield)	Large (≈ 300 nm in size)	Thicker (≈ 4.9 nm)
	Lower concentration (at C_i_ of 0.08 mg mL^–1^)	Higher (≈ 0.46% yield)	Small (≈ 20 nm in size)	Thinner (≈ 1.5 nm)
Centrifugal speed^204^	Higher speed (at ≥6,000 rpm)	Lower exfoliation yield	Small (≈ 301 nm in lateral size)	Thinner (3–10 layers)
	Lower speed (at 0–1500 rpm)	Higher exfoliation yield	Small (≈ 1,106 nm in lateral size)	Thicker (>10 layers)

## 3 Electrochemical Applications of the As-Exfoliated Materials

Investigations into the electrochemical behavior of TMDs offer significant potential for advancing their development across various electrochemical applications. While there exists a substantial body of literature dedicated to reviewing the applications of TMDs, particularly in areas such as hydrogen production,^29,205−210^ supercapacitors,^29,211^ and batteries,^209^ there is also a comprehensive review by Chia et al. that has focused on the fundamental electrochemical properties of TMDs, including MoS_2_, WS_2_, MoSe_2_, and WSe_2_.^212^ They underscored the significance of these materials possessing well-defined redox properties and explored their potential as electrode-modifier materials in key electrochemical applications. However, there has been limited research examining the influence of TMDs’ structural characteristics, including dimensions and crystal arrangement, on their electrochemical properties. Yet, the electrochemical behavior of 2D materials is known to be sensitive to their structural features, such as lateral size and defects, which can be controlled during synthesis. Therefore, understanding the interplay between structural and electrochemical properties of exfoliated flakes, including lateral size and thickness, is crucial. “The selection of suitable materials is paramount for optimizing electrochemical performance.” In the realm of electrochemistry, our focus will primarily revolve around three main applications: energy storage, ionic sieving membranes, and energy conversion, while exploring the impact of 2D materials’ structure on these applications.

### 3.1 Energy Storage Applications

Graphene has been recognized as one of the candidate materials among 2D materials. This is because graphene exhibits a high specific surface area (up to 2629 m^2^ g^–1^), once cleaved from graphite to a lower dimension. This aids the physical adsorption of electrolytes on the graphene sample. It is known that the more active surface area should improve the capacitance by enhancing the Helmholtz capacitance on the interfaces.^213^ Thus, the preparation of high quality (also refer to high surface area) is then important to further develop high performance energy storage. As mentioned in the synthesis section, the LPE technique can produce a scalable amount of 2D materials, while retaining the flake quality. Deerattrakul et al.^222^ demonstrated that the capacitance of graphene is relied on the flake dimension. The lower flake dimension appears to show high capacitance due to the increase in edge sites. The report shows that graphene with the lateral size of 100 nm exhibits the capacitance of 6.52 F g^–1^, increasing the lateral size to 1 μm, minimizing the capacitance to 0.53 F g^–1^. The work mentioned here is for pristine (ultralow oxygen content) graphene not “graphene oxide” or “reduced graphene oxide”. This is due to graphene oxide and its derivatives, which can provide high pseudocapacitive properties; however, the electrochemical performance depends on the doped species and its concentration.^214^ It is obvious that graphene itself cannot be an outstanding electrode material for energy storage if it was standalone. However, graphene shows predominant properties for composited materials, especially graphene composited with metal oxide materials (also referred to as layered materials), which exhibit a high ion intercalation degree.^32^ Moreover, graphene itself acts as an electrode binder, which can replace a traditional polymeric binder. This is due to its sheetlike structure with the van der Waals interaction behaving as a sticky glue when applied to active materials.^215^ This technique can be used to form a freestanding electrode, with detailed concepts reported by Xu and Gogotsi.^215^ With a variety of flake size/dimensions possibly controlled by the exfoliation and separation procedure, applying different graphene flake sizes to the composite materials could alter the capacitive deionization properties. Hirunpinyopas et al. found that an appropriate flake size of graphene binder should be *ca*. 350 nm (lateral length). Too large of flakes can diminish the electrical conductivity of the graphene while using too small of flakes could block the porosity of the electrode materials.^216^ Transition metal dichalcogenides with the structure analogous to graphene could also be applied in the same perspectives. A more beneficial thing that we could gain from using TMDs materials is the layer structure. The existing galleries can accommodate the ions via electrochemical intercalation, boosting the capacitance (also refer to capacity in batteries).^217^ Electrochemical intercalation of a variety of WSe_2_ flake sizes were studied, and it is found that the smaller in flake size exhibits high pseudocapacitive properties, including surface redox and intercalation.^204^ Bissett et al. demonstrated that the MoS_2_/graphene composited can enhance the overall capacitance of the prepared electrodes.^218^ Also, Nualchimplee et al. also reported that the selection of exfoliation solvent can affect the electrochemistry of the as-obtained materials, i.e., NMP/water can create an auto-oxidation of MoS_2_ to MoO_3_, which enlarged the interlayer between adjacent sheets leading to more ion accommodation during the charging process.^219^ These are some advantages of the understanding of exfoliation procedure of TMDs.

### 3.2 Ionic Sieving Applications

The increase in energy consumption, growth, and rapid industrialization and a shortage of sanitary water have become one of the 17 sustainable development goals (SDGs). To achieve these goals, membrane-based filtration technologies have been demonstrated to be favored over other purification processes (i.e., distillation and evaporation) due to energy efficiency and facile technology.^33,201,220^ Polymer-based membranes have been widely used for purification, but they still have drawbacks such as lack of high temperature resistances, low stability in harsh acid/base conditions and organic solvents, and low electronic conductivity for electrodialysis technologies. Two dimensional (2D) materials-based membranes have been demonstrated as novel membranes for water purification. This is due to the promising properties, i.e., physical sieving/adsorption and separation of unwanted molecules/ions, especially for seawater desalination.^33,201^ The membrane can be prepared by self-assembly techniques of the 2D material dispersions via vacuum and pressure filtrations. This provides the stacking of individual nanosheets to be formed as assembled 2D materials laminates or 2D laminar membranes. The mechanism of the membrane filtrations is based on (i) the effect of size exclusion obtained from nanochannels forming between nanosheets stacking and adjacent nanosheets and (ii) electrostatic interaction between charged materials and charged ionic/molecules which can be electrostatic attraction and repulsion. Nair et al. first demonstrated graphene oxide (GO) membranes for studying a variety of species with different hydrated diameter (i.e., ions, molecules, and gas) transport through GO membranes.^221^ It was found that the membrane can potentially block small solutes with the hydrated diameter cutoff ∼9 Å. This is because the membrane exhibited a network of nanocapillary channels that can generate a capillary-like pressure to allow smaller solutes transport through the membranes.

**Figure 12:**
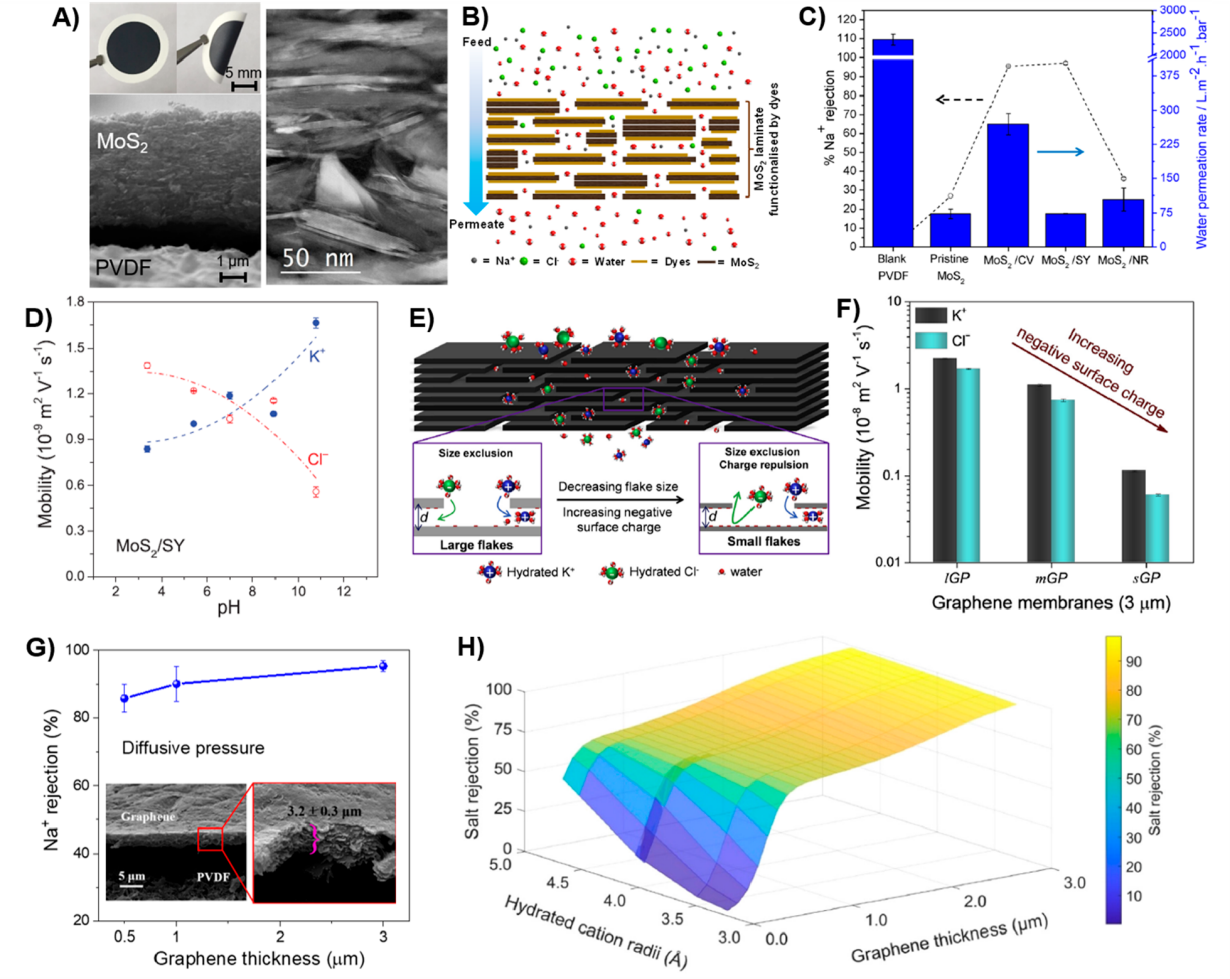
Ionic sieving applications. (a) Photographs, SEM, and HAADF TEM images of the chemical functionalized MoS_2_ membranes as well as (b) schematic showing the dye-functionalized MoS_2_ membranes. (c) Comparing membrane performances of various dye-functionalized MoS_2_ and pristine MoS_2_ membranes. Figure 12a–c: Reprinted from ref (220). Copyright 2017 American Chemical Society. (d) Potential dependent ionic sieving through dye-functionalized MoS_2_ membranes showing high cation selectivity (80%) at alkaline solutions. Reprinted under the terms of the CC-BY 3.0 license from ref (226). Copyright 2020 IOP Publishing. (e) Schematic laminar graphene membranes with different nanosheet sizes showing the effect of size exclusion and electrostatic interactions and (f) K^+^ and Cl^–^ mobility through graphene membranes indicating the suppression of ion mobility and the reduction of Cl^–^ mobility. Figure 12e,f: Reprinted with permission from ref (201). Copyright 2020 Elsevier. (g) Percentage of sodium ion rejection (Na^+^%) as a function of graphene thicknesses. Inset shows ultrahigh stability of laminar-stacked graphene membranes after being immersed in various salt concentrated solution, seawater, and different pH solutions. (h) Machine learning prediction for studying ion permeability through graphene membranes based on hydrated cation radii and the thickness of graphene membranes. Figure 12g,h: Reprinted with permission from ref (33). Copyright 2023 Royal Society of Chemistry.

However, the GO-based membranes still have drawbacks for use in water purification, for example, low resistance to strong acidic/alkaline solutions, lack of mechanical strength, and especially instability in aqueous solutions. The latter reasons clearly impact water-based applications due to the fact that the membranes cannot remain intact when readily swollen in aqueous solution.^33,201,222^ To minimize the swelling GO membranes, there were many reports with regard to this such as physical confinement^223^ and chemical reactions (i.e., cross-linking, and chemical reduction^224,225^). In fact, these methods are still in limited use in real-world industrial applications because of their unstable long-term uses as well as lack of large-scale production. Therefore, alternative 2D materials such as transition metal dichalcogenides (TMDs; MoS_2_ and WS_2_) and transition metal carbides/nitrides (MXene) can be prepared as laminar membranes which were suitable for applications in water purifications.^220,226,227^ This is due to TMDs-based membranes, which exhibited excellent performances with high stability in aqueous solution as well as under harsh pH conditions.^220,228^ However, the pristine TMDs membranes i.e., the MoS_2_ membrane, still have limitations in terms of water permeation rate and salt rejection which are the most important factors for filtration applications. To overcome this problem, Hirunpinyopas et al. demonstrated a facile chemical functionalization reaction on the MoS_2_ membranes using charged dye molecules^220^ as shown in Figure 12a,b. It was found that the functionalized MoS_2_ membranes can reject ∼99% of the sodium ion, while providing a water flow rate 5 times higher than previously reported GO-based membranes (Figure 12c). Also, the membranes can potentially maintain the laminar structure (no swelling) when kept in water for a long period of time (>6 months) and various organic solvents, indicating long-term stability. The enhanced permeability performance of the functionalized membranes results from not only the ion rejection from the critical channel width but also the change in surface charge properties of the membrane. The functionalization technique can improve the properties of the membrane interface, at which the ion permeability performance is significantly tuned. To understand the mechanism behind the effect of dye functionalization, Hirunpinyopas et al. studied potential dependent ionic sieving through the dye functionalized MoS_2_ membranes under different pH, ionic solute concentration, and ionic charge/size conditions.^226^ They found that the reduction in ion permeability through the dye functionalized MoS_2_ membranes was far better than that of the pristine one as well as other 2D materials-based membranes such as GO and MXene membranes. This can be explained by the fact that the surface chemistry of MoS_2_ membrane was controlled by the role of chemical functionalization, at which the cation selectivity can be boosted up to ∼80% under basic solutions as shown in Figure 12d. This work not only disclosed the ionic sieving mechanism of laminar stacking membranes but also was potentially applied in electro-dialysis and ion exchange for water purification technologies.

To fulfill the understanding of ionic sieving of 2D materials-based membranes, Hirunpinyopas et al. further demonstrated the effect of dimensions of graphene nanosheets (different lateral length and thickness) toward the ion and water permeability performance^201^ (Figure 12e,f). They found that the formation of laminar structure inside the membrane plays a crucial role in ion transport. They demonstrated that large and thick nanosheets would provide well-uniformed laminar structure giving a smooth pathway while small and thin nanosheets could make complex stacked nanosheets leading to tortuous nanocapillary channels. Thus, the ionic sieving properties can be tuned by using the different nanosheet sizes, in which the smaller nanosheets with high negative charge can enhance both physical sieving and charge repulsion as shown in Figure 12f. Moreover, to make highly stable membranes with low cost, Paechotrattanakul et al. recently demonstrated graphene membranes prepared by graphene nanosheets possessing ultralow oxygen contents (∼1 at %), unlike GO and rGO synthesis (>30 at % oxygen).^33^ The graphene dispersion in this work was prepared via a sonication-assisted exfoliation, which this technique still retains the graphitization (low defect formation) while the chemical process known as Hummer’s method was widely used for a case of GO synthesis. They found that the as-prepared graphene membranes showed ultrahigh stable membranes with no swelling and disintegration when immersed in various aqueous solutions, such as synthetic seawater, at various pH values for a long period of time. The membranes can reject small ionic solutes and different charged dye molecules, which the ionic and molecular sieving mechanism was based on the effect of size exclusion and electrostatic repulsion as shown in Figure 12g,h. The membrane performances were also investigated using a machine learning study to optimize the membrane for future water filtration technologies (Figure 12h).

### 3.3 Energy Conversion Applications

**Figure 13:**
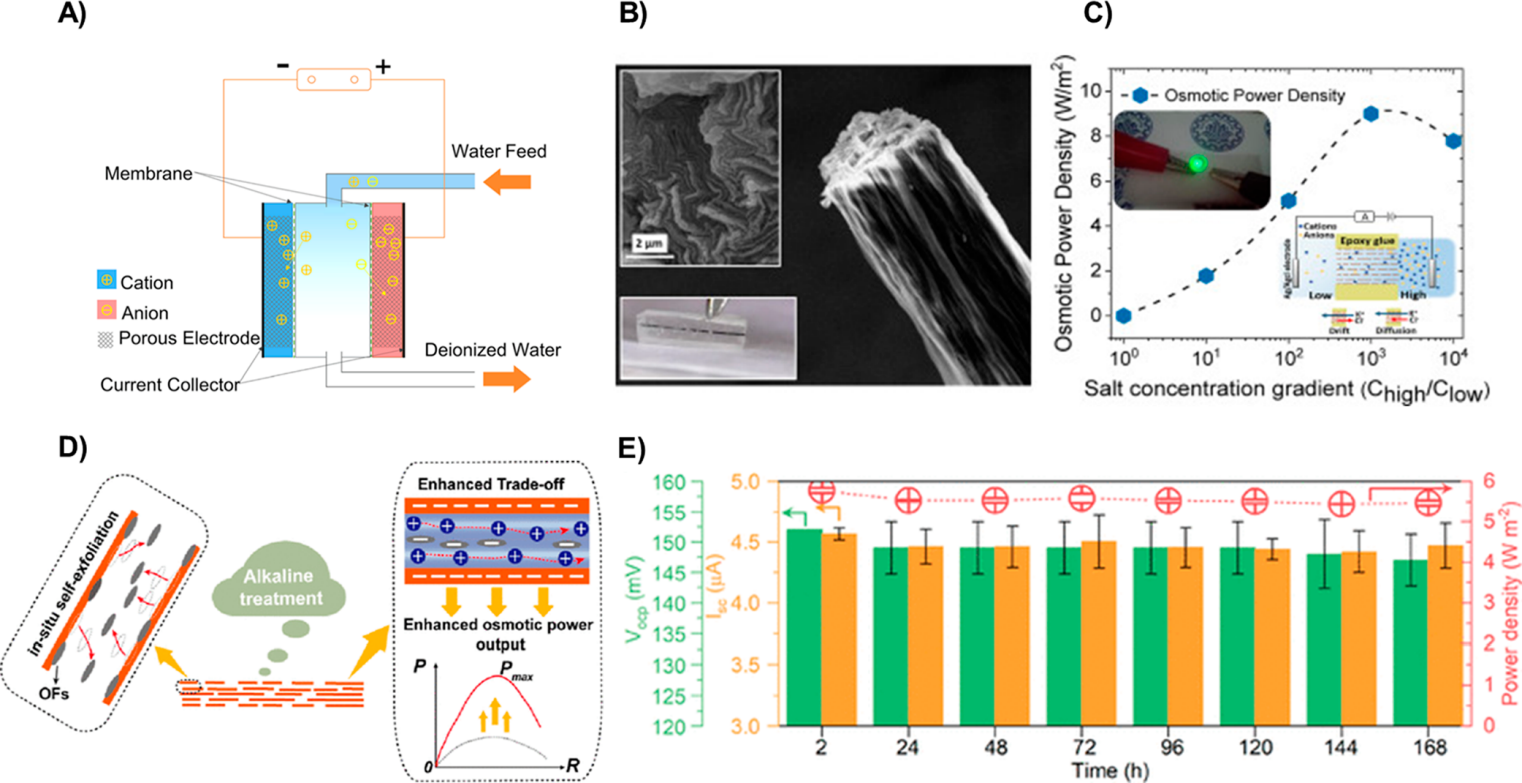
Energy conversion applications. (a) Schematic showing capacitive deionization (CDI) process consisting of semipermeable porous membranes, power supply, and salt electrolyte. Reprinted with permission from ref (231). Copyright 2017 AIP Publishing. (b) Fiberlike GO membrane for energy conversion and (c) osmotic power density of fiberlike GO membrane as a function of salt concentration gradients. Figure 13b,c: Reprinted with permission from ref (244). Copyright 2020 Elsevier. (d) Schematic showing the GO membrane stabilized by alkaline treatment for producing osmotic power. (e) Corresponding output power density of the GO membrane within a long-term (7 days) test. Figure 13d,e: Reprinted from ref (243). Copyright 2022 American Chemical Society.

Bridging between energy storage and ionic sieving applications as aforementioned above, capacitive deionization (CDI) is one of the water purification technologies that could be applied with laminar 2D materials-based membranes.^229,230^ Such technology is widely known as an electrochemical water filtration process that not only stores the energy as a capacitive property but also sieves ionic solutes as a seawater desalination. This indicated the capability of the CDI system that can deliver clean water and energy storage simultaneously as shown in Figure 13a.^231^ In general, there are two steps for the CDI operation that is similar to a capacitor system: (i) ion adsorption and (ii) ion desorption. For the first step, the charged ionic solutes were adsorbed on the positively and negatively charged porous electrode pairs via a charging step (i.e., electro-sorption step). This can provide low concentration of the ionic electrolyte system and more purified water under consecutive electrodes or the flow electrode cell. Then, the absorbed ionic species can be released from the electrodes which can provide the energy known as a discharged process. This step can regenerate the electrodes which can be potentially used for long-term cycles.^230,232^ Due to the electrode properties playing an important role in the CDI performances, the electrode materials must be considered to acquire high CDI performances, e.g., materials should consist of conductivity, high surface area, and particularly high stability in high salt concentrated solutions. To explore the promising materials, the use of 2D material-based membranes have been introduced as electrode materials for the CDI system, which could be of interest for future commercial CDI membranes. There are many examples of the use of TMD materials for the CDI process.^233−235^ Xing et al. used exfoliated MoS_2_ as an electrode material for the CDI process.^233^ They found that MoS_2_ nanosheets exhibited excellent ion mass adsorption (∼8.8 mg g^–1^) and ion volume adsorption (∼16.5 mg cm^–3^) using 400 mM NaCl solution with an applied potential of 1.2 V. The CDI performance obtained was better than that of bulk MoS_2_ materials and the typical CDI electrode (carbon-based materials; activated carbon) even at a low salt concentration (50 mM).

Apart from seawater desalination of the semipermeable membranes, 2D material-based membranes can also be applied to harvest clean energy from the salinity gradient. The obtained energy known as osmotic energy or “blue energy” was produced from the chemical potential difference between high concentrated solution (seawater) and low concentrated solution (fresh water). This application is defined as a clean, abundant, and sustainable source of energy. The technique can be applied to produce energy naturally at estuaries that are a boundary between seawater and river.^236−238^ However, one of the core technologies for blue energy harvesting is still limited from conventional semipermeable membrane performances. To overcome this key challenge, a novel class of membranes should be considered to enhance size and charge ion selectivity with high water permeance leading to large-scale blue energy conversion.^236,239^ Recently, the semipermeable 2D material-based membranes in the form of single layer membranes and laminar stacked membranes have been introduced as sustainable power generators.^237,240−244^ The use of exfoliated 2D materials as membranes for harvesting energy has been reported. Ghanbari and Esfandiar demonstrated fiber-like GO membranes to produce energy from salt concentration gradients.^244^ The stability of the GO membrane was controlled using cation intercalations to confine their laminar structure as shown in Figure 13b,c. The as-obtained GO membrane provided high osmotic power of up to 38 W m^–2^. Recently, Qian et al. prepared stable GO membranes using the self-exfoliation process from oxidative fragments of GO under basic solution.^243^ The properties of as-obtained GO membrane can be tuned between ion selectivity and permeability, which can boost the osmotic power up to 38 W m^–2^ with a cation selectivity of 0.9 under long-term tests (7 days) as shown in Figure 13d,e. In addition to graphene and its derivatives, Zhu et al. showed that metallic 2D MoS_2_ composites can serve as efficient osmotic power generators.^245^ Their study and computational modeling demonstrated that the increased electron density of metallic MoS_2_ can enhance the attraction of cations to the surface, leading to excellent ion selectivity and high ionic flux, thereby facilitating transmembrane ion diffusion. When natural river water and seawater are combined, the power density can reach approximately 6.7 W m^–2^. Therefore, the use of advanced 2D materials in the form of scalable laminar membranes for nanofluidic devices could be applied to ionic and molecular sieving as well as energy harvesting.

The following table outlines the influence of 2D nanosheet size on electrochemical applications, providing enhanced clarity. In addition to properties like thickness and dimensions, the polymorphism of these materials significantly affects their electrochemical properties. During the exfoliation process, particularly during Li-ion intercalation, 2D materials, especially TMDs, may undergo phase transitions from the 2H-phase (with a trigonal prismatic structure) to the 1T-phase (with an octahedral structure).^121,129^ This transition occurs with the introduction of excess charge, causing a transformation in the unit cell structure from trigonal prismatic to octahedral. Such transitions can lead to significant alterations in electrochemical properties, as the original 2H-phase loses its semiconducting properties while the constructed 1T-phase gains metallic properties, ultimately influencing performance in various electrochemical applications.

For instance, 1T-MoS_2_ nanosheets exhibit enhanced electrocatalytic activity for the hydrogen evolution reaction (HER) compared to 2H-MoS_2_ and are efficient materials for supercapacitor applications.^130^ Additionally, Acerce et al. demonstrated that exfoliated MoS_2_ nanosheets with a high concentration of the metallic 1T phase can achieve capacitance values ranging from ∼400 to ∼700 F cm^–3^ in various aqueous electrolytes.^130^ They also showed that this material is suitable for high-voltage (3.5 V) operation in nonaqueous organic electrolytes, displaying superior volumetric energy and power density values. Coulombic efficiencies exceed 95%, and stability over 5000 cycles. These favorable electrochemical properties of 1T-MoS_2_ layers are primarily attributed to their hydrophilicity and high electrical conductivity. Beyond energy storage applications, the phase transition from 2H to 1T also affects performance in water-permeable membranes. Hu et al. demonstrated that water and ion permeation through 1T-MoS_2_ membranes exhibit pH-dependent hysteresis with permeation rates switching by several orders of magnitude.^246^ They attributed this phenomenon to the unique characteristics of the 1T phase of MoS_2_, including surface charge and exchangeable ions on the surface.

Nevertheless, despite their exceptional electrochemical performance, 1T-phase materials have drawbacks related to their stability and sensitivity to ambient conditions.^121,247,248^ Essentially, they demonstrate reduced thermodynamic stability when contrasted with the 2H phase.^121,129^ Consequently, these materials tend to favor the 2H phase, and even if they are in the 1T phase, they commonly revert back to the 2H phase within a few weeks due to the metastable nature of the transformed 1T-TMDs (see Table 2).^121^

**Table 2:** Influence of the Size of Exfoliated 2D Material Nanosheets on Different Electrochemical Applications^a^

Electrochemical applications	2D Materials	Flake dimensions	Performance
Energy storage and conversion applications	Graphene^222^	Small (100 nm in lateral size)	High capacitance (6.52 F g^–1^)
		Large (1 μm in lateral size)	Low capacitance (0.53 F g^–1^)
	WSe_2_^204^	Small (≈ 301 nm in lateral size)	Lower overpotential value for HER performance (−400 mV)
		Large (≈ 1,106 nm in lateral size)	Higher overpotential value for HER performance (−929 mV)
Ionic sieving applications	Graphene^201^	Smaller flakes	Complexed stacked nanosheets, tortuous capillary channels
		Larger flakes	Uniformed laminar stacking, giving smooth pathways

a The benchmark is based on no additives added to the electrode.

## 4 Advancement of 2D Materials Research with Data Science and Machine Learning

### 4.1 Introducing Applications of Data Science and Machine Learning in 2D Materials

The abundance of research data in this age of technology, which is a requirement for effective machine learning,^249,250^ is astronomical compared to the past, where access to scientific journals and the Internet were not common. Therefore, utilization of data science and machine learning has emerged as a promising tool to investigate insights and underlying mechanisms in a wide-range of scientific communities. Another reason for the large advancement in data science and machine learning is easier data acquisition methods. The collection of data has been streamlined by various modern techniques, for example, web-scraping^251,252^ and LLM assisted data collection (e.g., battery materials data set collected via natural language processing toolkit ChemDataExtractor^253^ by Huang and Cole). Additionally, simulation and theoretical data are easier to obtain due to increased computational power (Moore’s law^254^). This data is then compiled for use in data science and machine learning research (e.g., TMDs data set calculated via density functional theory^255^ by Muller et al.). Lastly, manually gathering data from literature to establish data sets is also prominent with easier access to journals (e.g., Yuan et al. carbon materials data set^256^). After the data is gathered, various techniques can be used to understand the synergies and discover new interactions between features and target, which ranges from simple data analysis techniques such as scatterplots and Pearson correlation heatmaps to advance machine learning algorithms which utilize feature importance analysis, such as Shapley Additive exPlanations (SHAP),^257^ feature permutation importance,^258,259^ and partial dependence plots.^260^ Therefore, the power of data is eminent to provide fruitful relation between affected features and key characteristics of interested systems within an economical time frame. This also allows maximum efficiency and usage of experimental data that would rather be “wasted” if not utilized.

Even though traditional data analysis techniques such as scatterplots and Pearson correlation heatmaps are essential to discover the synergies between 2 variables, there is difficulty in displaying the interaction of variables in more complex interactions (i.e., nonlinear,^261^ highly noisy^262^) in higher dimensional space (i.e., multivariate interaction^263^). Therefore, machine learning and feature importance analysis is utilized to discover the interactions of complex variables and simplifying the synergies for human understanding.^264^ In this review, the utilization of data science and machine learning in 2D materials research, namely in their use in energy storage, catalysts, and material properties and synthesis, is especially emphasized to provide a general idea for application of data science in the field of materials development. The key usage for data science and machine learning that will be explored is optimization and novel discovery. Optimization is the process of discovering the most optimal conditions for material synthesis. For example, the exfoliation of transitional metal dichalcogenides (TMDs), which can include the interactions between centrifugal speed, sonication duration, surfactant concentration, and more, can be handled by machine learning models suited for high-dimensional and complex data interactions. After training the model, feature importance analysis techniques can be used to find the optimal conditions to obtain the highest yield. For novel discovery, important molecular descriptors can be used for machine learning, which can be calculated from easily accessible cheminformatics toolkits such as RDKit.^265^ These molecular descriptors provide information such as molecular weight (0D), molecular formulas (1D), bonds (2D), spatial orientation (3D), and molecular dynamics (4D). Coupled with experimental data for a target value such as capacitance and the structure of the electrode or electrolyte, machine learning can aid in finding the most important molecular descriptor which is needed for high capacitance, accelerating the discovery and synthesis of novel 2D materials. A part of novel discovery is clustering, which uses unsupervised machine learning models (e.g., DBSCAN^266^) to cluster and group 2D material data with similar properties using molecular descriptors or collected features. This aids in grouping materials with specific molecular descriptors that have useful properties (i.e., high electrical conductivity, surface area) to pave a way for synthesis of desired 2D materials.

### 4.2 Data Science and Machine Learning for Optimization and Novel Discovery of 2D Materials

Heteroatom-doped graphene is an example of 2D materials with outstanding electrochemical properties over pristine graphene, thus it is one of the candidates as electrode material for supercapacitor development. However, the underlying effects of its electrochemical features which relate to optimization of capacitance is not fully understood. Jitapunkul et al.^214^ proposed a simple approach based on Pearson correlation analysis, curve fitting, and scattered interpolation-extrapolation which apply to gathered experimental data to investigate effects of significant features related to capacitive boosting of heteroatom-doped graphene. The positive correlation between specific surface area and specific capacitance can be clearly noted for all single heteroatom-doped graphene, double heteroatom-doped graphene, and triple heteroatom-doped graphene, except for O/S codoped graphene which may be due to a limited number of gathered data. This suggested that the specific capacitance tends to increase along with the enlarged specific surface area. Interestingly, the negative correlation between structural defect (*I*_D_/*I*_G_) and specific capacitance is found for nitrogen-doped and oxygen-doped graphene which may be the result of poor electrical conductivity and inertia of oxygen and nitrogen-based functional groups.^267^ The capacitive retention can also be illustrated through exponential decay equation fitting of scattered current density versus specific capacitance which indicates the superiority in decay rate of heteroatom-doped graphene over the undoped one. More importantly, the optimum doping condition can be identified through scattered interpolation-extrapolation which suggest the percentage of heteroatom-doping on graphene as single, double, and triple atoms doping with oxygen, nitrogen, and sulfur as the main atomic species. Deshsorn et al.^268^ have utilized an ensemble technique so-called “stacking” to combine the benefits of multiple machine learning algorithms from two families which are regression-based and tree-based algorithms. This strategy tends to increase the prediction accuracy and generalization of the final model over the application of the standalone model. The main aim of this so-called “stacking model” is to provide the accurate capacitance prediction from various electrochemical features of heteroatom-doped graphene. However, the features selection is also crucial, therefore, Shapley values calculated from SHAP analysis is then employed to determine the significance of features. As a result, the top three important features can be determined from the top three ranked Shapley values which are amount of sulfur doping, specific surface area, and amount of nitrogen-doping, respectively. This indicates the importance of those features on the capacitive ability of heteroatom-doped graphene which is also significantly related to the prediction ability of the stacking model. Is is not only with the application in electrochemistry that graphene has been recognized as a potent material, but it has also gained recognition in catalytic application as well. Saeloo et al.^269^ has adopted a random forest algorithm to study the underlying relationship between electrocatalytic features of graphene support with decorated gold nanoparticles and output potential for hydrogen evolution reaction (HER). The random forest prediction model and SHAP analysis revealed the significance of gold nanoparticles loading and current density on the output potential which was directly related to the catalytic performance of the graphene support. Therefore, the gold nanoparticles loading onto the graphene support has possessed the superior catalytic performance over the unloaded one which exactly correlated to experimental data.

Since, transition metal dichalcogenides (TMDs) are another 2D material that exhibit outstanding chemical and physical properties. Hence, it is a promising material for the application of various devices. However, the relationship between physical features such as structural characteristics and the performance of final materials is still not fully understood through experimental approaches. Therefore, application of machine learning algorithms or data analysis should reveal the underlying relation between those physical features and the properties of TMDs. An example of using machine learning for novel discovery of TMDs is a study by Malakar et al.^270^ They utilized molecular dynamics simulation data of monolayer TMDs (M = Mo, W and X = S, Se) in a long–short-term memory network (LSTM) and feed forward neural network (FFNN) to predict the fracture stress, fracture strain, and Young’s modulus of TMDs. Their results show that LSTM, though more computationally complex than FFNN, predicted the stress–strain relationship and mechanical properties well by taking into account time-series in the model. With the utilization of this approach with more data, a preliminary selection of TMDs with desired properties can be obtained to reduce cost and time of experiments. Another challenge is the optimal synthesis condition (i.e., exfoliation parameters) of TMDs to acquire the highest yield. As an example, several conditions for exfoliation (e.g., sonication duration, sonication power, centrifugal speed) can be used as the features for a machine learning model to predict yield. Once the highest accuracy model is trained, feature importance analysis is then used to find the optimal conditions for the highest yield. This method hopes to escalate the quality and improve the design of TMDs synthesis in the future within an economical time and cost.

Transition metal carbide/nitride (Mxenes) are graphene-like 2D materials which have gained popularity during recent years, especially for application of energy storage due to its superb electrochemical properties. Rong et al.^271^ have used machine learning to predict the mechanical properties of MXene-based aerogels from 34 data sets of a Ti_3_C_2_ Mxene experiment. Artificial neural network, support vector machine, and random forest models have been utilized to investigate the synergistic relation between Mxene and nanocellulose aerogels which improve electrical conductivity. They have concluded that the artificial neural network provided the most accurate prediction over other adopted models. The maximum compression modulus was pinpointed to be 29 kPa, and the amount of Ti_3_C_2_ Mxene was a significant factor that directly related to compressive strength of the aerogel. Qian et al.^272^ have reported a review on the emerging trend of machine learning to study properties of Mxene which has drastically risen since 2015. Rajan et al.^273^ has adopted Kernel Ridge Regression (KRR), Support Vector Regression (SVR), Gaussian Process (GPR), and Bootstrap aggregating Regression algorithms to predict the bandgap of Mxenes. They found that the GPR model can provide the prediction with the highest accuracy. Therefore, with the help of a machine learning model, the stable Mxene structure can be identified based on the bandgap with a modest computational time. The catalytic, electrical, and optical properties of Mxene materials could be altered by a surface functional group which tends to be controllable. Unfortunately, the screening for structural interface defects and functional groups is not convenient and is time-consuming.^274^ Consequently, the virtual screening based on integration of first-principles simulation and machine learning can be used to efficiently investigate the surface functional groups and defects with a significantly shorter time frame. Frey et al.^275^ have proposed the quantum integration with a neural network information model to investigate the defects of 2D materials. They have utilized the developed model for identification of defects on various 2D materials which are of direct consequence and provide crucial information for Mxene synthesis design.

**Figure 14:**
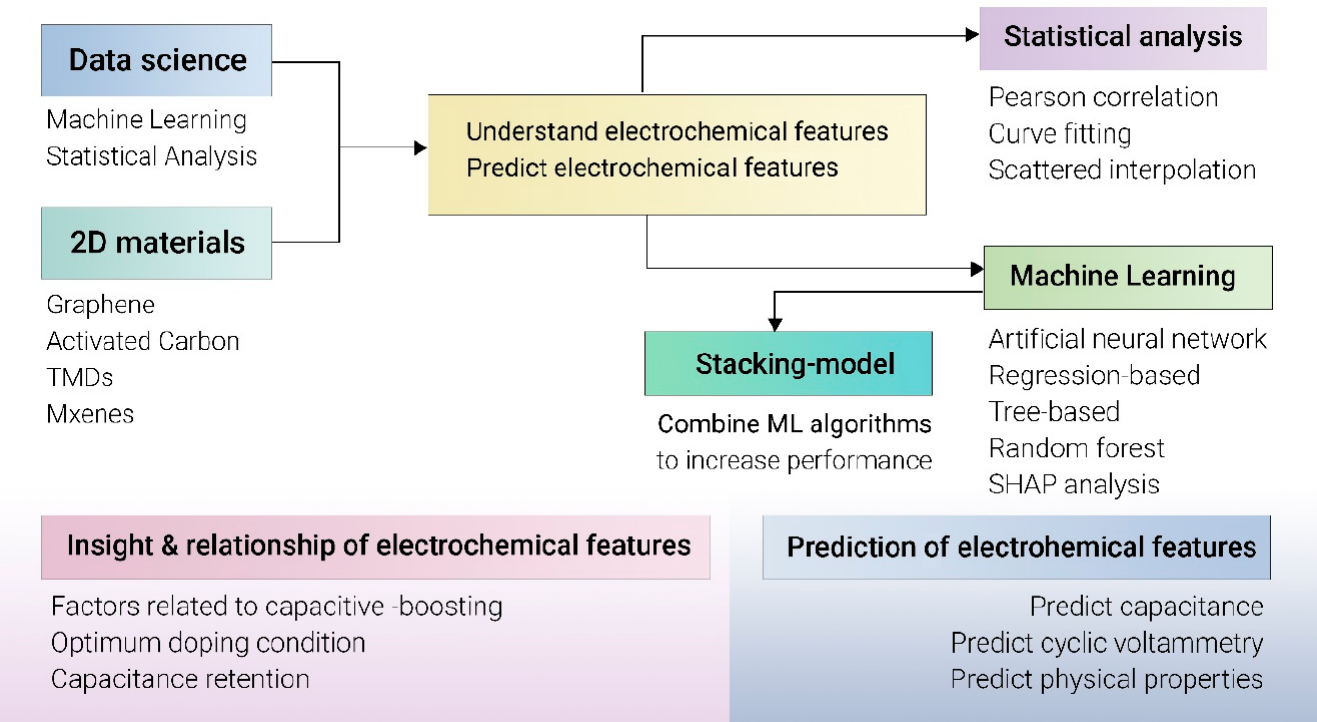
Workflow for application of data science to the study of 2D materials.

Thus, utilizing machine learning and data science in the field of 2D material research allows the optimization and discovery of novel materials. Furthermore, insights into the relationship of variables in higher dimensional space is simplified for human understanding, effectively increasing the interpretability of the synergies of variables. As a summary, the workflow for application of data science to study and predict the electrochemical, physical, or chemical features of 2D material is illustrated below as a general guideline (Figure 14).

## 5 Conclusion

Understanding the mechanism of sonication-assisted liquid phase exfoliation in transition metal dichalcogenides provides insights into the characteristics of the produced 2D nanosheets. An in-depth examination of exfoliation parameters, including sonication power and time, solvent and surfactant selection with their starting concentrations, and centrifugation speed, hence reveals the impact of each parameter on the resulting nanosheets for various applications, particularly in electrochemical applications. For instance, prolonged sonication may introduce defects and reduce the size of the nanosheets. Similarly, higher concentrations of surfactant and starting material can lead to restacking and the formation of alternative species, such as MoO_*x*_ from MoS_2_. Additionally, centrifugal speed significantly affects the lateral sizes and thickness of the nanosheets. Given the profound effects of these parameters on nanosheets, a detailed understanding of each step is crucial to minimize defects and achieve optimal exfoliation efficiency for 2D nanosheets. This pursuit of “highest efficiency” aims to produce high-quality nanosheets suitable for specific applications. Determining the required nanosheet quality necessitates consideration of the unique properties demanded by each application, enabling the identification of optimal conditions for each parameter.

For example, in ionic sieving membranes, nanosheet lateral length and thickness influence ion and water permeability. Smaller graphene flakes may result in complex stacked nanosheets, while larger and thicker nanosheets offer well-uniformed laminar stacking. In energy conversion, nanosheets need to be enlarged with enhanced charge ion selectivity and high water permeance for large-scale blue energy conversion. Conversely, in electrochemical applications, smaller WSe_2_ nanosheets are favored for energy storage due to their superior pseudocapacitive properties compared to larger ones, potentially requiring higher centrifugation speeds. In addition to the mentioned 2D TMDs and graphene, various emerging 2D materials, such as the synthetic route and electrochemical performance of SnS nanosheets in batteries, are equally significant and merit understanding.

Furthermore, in the realms of data analysis, the number of data sets used for data analysis and machine learning has increased in recent years due to novel methods for data collection. This includes data sets for material discovery and optimization of 2D materials. Various techniques such as SHAP, feature permutation importance, and partial dependence plots can be used to discover complex multivariate interactions too complex for human understanding. Additionally, data science and machine learning can be used as an aid for scientists to discover the optimal conditions for the exfoliation of TMDs for the highest yield.

## 6 Perspectives and Future Directions

Through a deep understanding of the fundamentals of the LPE process, we aim to develop optimized synthesis conditions capable of consistently producing 2D material nanosheets with ideal quality for further exploration of their underlying mechanisms and applications. Furthermore, it can be anticipated that with the integration of data science and machine learning, specific exfoliation conditions tailored to particular applications can be identified. The inclusion of data science and machine learning in the field of 2D material research is promising. From the literature, the year 2023 shows 7208 articles for the keywords “2D material machine learning” compared to 62 articles in 2001. This shows the increase in interest of machine learning enthusiasts and material scientists. By incorporating these fields, one can observe the advancement of 2D material research using the incredible calculation speed of today’s processors, which allows tackling of tasks beyond human capabilities using computers. This includes dealing with astronomical amounts of data, finding multidimensional relationships, and data mining in data archives too complex for human understanding. Not only does increasing calculation power provide benefits to machine learning, it also provides benefits to simulation work, which can be used as the input of many machine learning models. Another important point is wastage of data and the emphasis of reduction of chemical wastes. By repurposing experimental data from past research into a training set for machine learning models, the reduction of cost, time, and environmental impact is achieved without doing repeat experiments. Overall, the inclusion of advanced techniques in data science and machine learning allows humans to discover useful patterns hidden inside complex data, reduce chemical wastes and costs from repeat experiments, and accelerate the discovery and optimization of 2D materials.
